# Differential microRNA editing may drive target pathway switching in human temporal lobe epilepsy

**DOI:** 10.1093/braincomms/fcad355

**Published:** 2024-01-03

**Authors:** Kelvin E How Lau, Ngoc T Nguyen, Jaideep C Kesavan, Elena Langa, Kevin Fanning, Gary P Brennan, Amaya Sanz-Rodriguez, Javier Villegas-Salmerón, Yan Yan, Morten T Venø, James D Mills, Felix Rosenow, Sebastian Bauer, Jørgen Kjems, David C Henshall

**Affiliations:** Department of Physiology and Medical Physics, Royal College of Surgeons in Ireland University of Medicine and Health Sciences, Dublin D02 YN77, Ireland; FutureNeuro SFI Research Centre, Royal College of Surgeons in Ireland University of Medicine and Health Sciences, Dublin D02 YN77, Ireland; Department of Physiology and Medical Physics, Royal College of Surgeons in Ireland University of Medicine and Health Sciences, Dublin D02 YN77, Ireland; FutureNeuro SFI Research Centre, Royal College of Surgeons in Ireland University of Medicine and Health Sciences, Dublin D02 YN77, Ireland; Department of Physiology and Medical Physics, Royal College of Surgeons in Ireland University of Medicine and Health Sciences, Dublin D02 YN77, Ireland; FutureNeuro SFI Research Centre, Royal College of Surgeons in Ireland University of Medicine and Health Sciences, Dublin D02 YN77, Ireland; Department of Physiology and Medical Physics, Royal College of Surgeons in Ireland University of Medicine and Health Sciences, Dublin D02 YN77, Ireland; FutureNeuro SFI Research Centre, Royal College of Surgeons in Ireland University of Medicine and Health Sciences, Dublin D02 YN77, Ireland; Department of Physiology and Medical Physics, Royal College of Surgeons in Ireland University of Medicine and Health Sciences, Dublin D02 YN77, Ireland; FutureNeuro SFI Research Centre, Royal College of Surgeons in Ireland University of Medicine and Health Sciences, Dublin D02 YN77, Ireland; FutureNeuro SFI Research Centre, Royal College of Surgeons in Ireland University of Medicine and Health Sciences, Dublin D02 YN77, Ireland; UCD School of Biomolecular and Biomedical Science, UCD Conway Institute, University College Dublin, Dublin 4, Ireland; Department of Physiology and Medical Physics, Royal College of Surgeons in Ireland University of Medicine and Health Sciences, Dublin D02 YN77, Ireland; FutureNeuro SFI Research Centre, Royal College of Surgeons in Ireland University of Medicine and Health Sciences, Dublin D02 YN77, Ireland; Department of Physiology and Medical Physics, Royal College of Surgeons in Ireland University of Medicine and Health Sciences, Dublin D02 YN77, Ireland; FutureNeuro SFI Research Centre, Royal College of Surgeons in Ireland University of Medicine and Health Sciences, Dublin D02 YN77, Ireland; The SFI Centre for Research Training in Genomics Data Science, University of Galway, Galway H91 TK33, Ireland; Omiics ApS, 8200 Aarhus N, Denmark; Interdisciplinary Nanoscience Centre (iNANO), Department of Molecular Biology and Genetics, Aarhus University, Aarhus 8000, Denmark; Omiics ApS, 8200 Aarhus N, Denmark; Interdisciplinary Nanoscience Centre (iNANO), Department of Molecular Biology and Genetics, Aarhus University, Aarhus 8000, Denmark; Department of Clinical and Experimental Epilepsy, Queen Square Institute of Neurology, University College London, London WC1N 3BG, United Kingdom; Chalfont Centre for Epilepsy, Chalfont St.Peter SL9 0RJ, UK; Department of (Neuro)Pathology, Amsterdam Neuroscience, Amsterdam UMC, University of Amsterdam, 1105 AZ Amsterdam, The Netherlands; Goethe-University Frankfurt, Epilepsy Center Frankfurt Rhine-Main, Department of Neurology, University Hospital, 60590 Frankfurt, Germany; Goethe-University Frankfurt, LOEWE Center for Personalized Translational Epilepsy Research (CePTER), 60590 Frankfurt, Germany; Goethe-University Frankfurt, Epilepsy Center Frankfurt Rhine-Main, Department of Neurology, University Hospital, 60590 Frankfurt, Germany; Goethe-University Frankfurt, LOEWE Center for Personalized Translational Epilepsy Research (CePTER), 60590 Frankfurt, Germany; Interdisciplinary Nanoscience Centre (iNANO), Department of Molecular Biology and Genetics, Aarhus University, Aarhus 8000, Denmark; Department of Physiology and Medical Physics, Royal College of Surgeons in Ireland University of Medicine and Health Sciences, Dublin D02 YN77, Ireland; FutureNeuro SFI Research Centre, Royal College of Surgeons in Ireland University of Medicine and Health Sciences, Dublin D02 YN77, Ireland

**Keywords:** RNA editing, epigenetics, hippocampus, noncoding RNA, seizure

## Abstract

MicroRNAs have emerged as important regulators of the gene expression landscape in temporal lobe epilepsy. The mechanisms that control microRNA levels and influence target choice remain, however, poorly understood. RNA editing is a post-transcriptional mechanism mediated by the adenosine acting on RNA (ADAR) family of proteins that introduces base modification that diversifies the gene expression landscape. RNA editing has been studied for the mRNA landscape but the extent to which microRNA editing occurs in human temporal lobe epilepsy is unknown. Here, we used small RNA-sequencing data to characterize the identity and extent of microRNA editing in human temporal lobe epilepsy brain samples. This detected low-to-high editing in over 40 of the identified microRNAs. Among microRNA exhibiting the highest editing was miR-376a-3p, which was edited in the seed region and this was predicted to significantly change the target pool. The edited form was expressed at lower levels in human temporal lobe epilepsy samples. We modelled the shift in editing levels of miR-376a-3p in human-induced pluripotent stem cell-derived neurons. Reducing levels of the edited form of miR-376a-3p using antisense oligonucleotides resulted in extensive gene expression changes, including upregulation of mitochondrial and metabolism-associated pathways. Together, these results show that differential editing of microRNAs may re-direct targeting and result in altered functions relevant to the pathophysiology of temporal lobe epilepsy and perhaps other disorders of neuronal hyperexcitability.

## Teaser

Lau *et al*. used RNA-sequencing data to characterize the microRNA editing landscape of human temporal lobe epilepsy, finding lower editing of miR-376a-3p. Modelling this reduction using antisense oligonucleotides upregulated mitochondrial pathway genes in neurons. The findings suggest roles for small RNA editing in the molecular pathophysiology of epilepsy.

## Graphical Abstract

**Graphical abstract:**
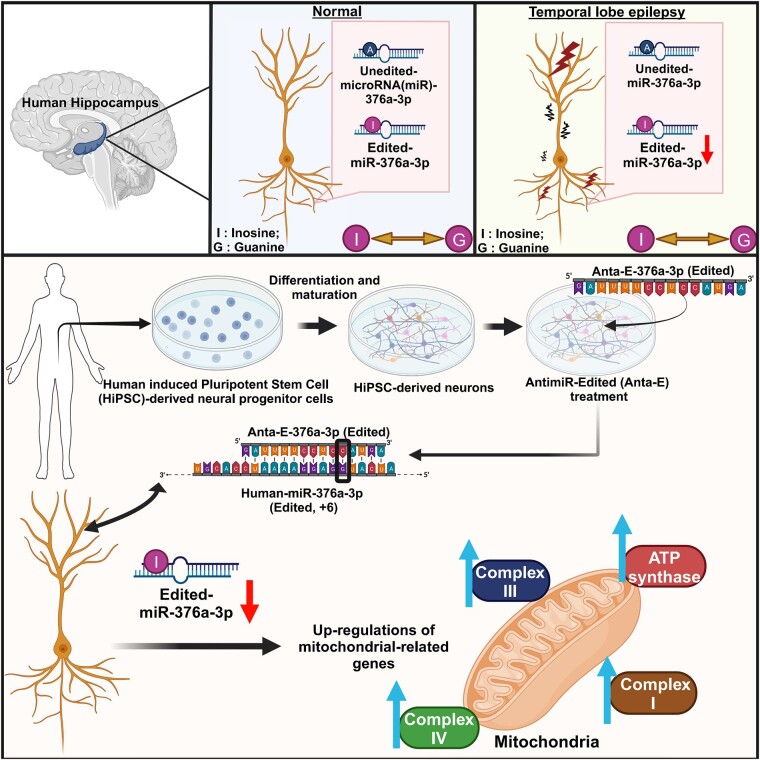

## Introduction

RNA editing is an essential post-transcriptional modification in which nucleotides within an RNA moiety are subjected to base substitutions, deletions, insertions or covalent modification.^1^ In humans, the adenosine deaminase acting on RNA (ADAR) family of enzymes mediates the most common form of editing, namely the deamination of adenosine to inosine (A-to-I) which is later interpreted as guanosine by the translational and splicing machinery, and can lead to changes to the encoded amino acid.^1-3^ Mammals possess three subtypes of ADAR enzymes—ADAR1 (*ADAR*), ADAR2 (*ADARB1*) and ADAR3 (*ADARB2*). ADAR1 and ADAR2 are active deaminases that are expressed in multiple body tissues.^4^ ADAR3 is expressed exclusively in the brain, although its substrates and functions are poorly understood.^5,6^ ADAR-mediated RNA editing has been shown to diversify protein-coding transcripts in eukaryotes, control innate immune responses and modulate neural activity.^1,4^ RNA editing is essential for brain development and function. For example, editing at the Q/R site of glutamate ionotropic receptor AMPA type subunit 2 (GluA2) by ADAR2 affects trafficking and limits calcium permeability, with reduced GluA2 editing promoting excitotoxicity and seizures.^7^ Dysregulation of A-to-I editing has been associated with several brain diseases.^8,9^

Epilepsy is a common, chronic neurologic disorder characterized by recurring spontaneous seizures.^10^ Temporal lobe epilepsy (TLE) is the most common form of treatment-resistant epilepsy in adults.^11^ Analysis of brain tissue obtained after surgical treatment of refractory epilepsy, as well as from animal models of TLE, reveals multi-pathway cellular changes including neuron loss, gliosis, inflammation and reorganization of connections and extracellular structures.^12,13^ Notably, differential editing was detected in 256 protein-coding transcripts in hippocampal samples from an experimental model of TLE.^14^

Recent studies have suggested a crucial role for microRNAs (miRNAs) in the development and progression of TLE.^15^ MiRNAs are short (∼22 nucleotides) noncoding RNAs that post-transcriptionally control gene expression through base-pairing within the 3’ untranslated region of messenger RNAs (mRNAs), leading to target degradation or translational inhibition.^16^ In the mammalian brain, miRNA play important roles in determining the excitable properties of neurons by regulating synaptic structures,^17^ glial function and inflammatory responses to injury.^18^ Dysregulation of miRNAs has been observed in brain tissue from epilepsy patients, and the targeting of miRNAs using antisense oligonucleotides (antimirs) has been reported to reduce seizures in animal models of TLE.^15,19^

ADARs also act on noncoding RNAs such as the precursor forms of miRNAs.^16,20-22^ Editing of miRNAs can impact on processing and redirection of the target mRNAs, leading to de-repression of some targets and engagement of new, previously untargeted transcripts.^16^ The extent to which miRNAs are edited in human TLE is unknown but could have implications for understanding how miRNAs control gene expression in epilepsy and the development of diagnostic biomarkers or therapeutics. Here, using small RNA-sequencing data,^23^ we characterize the extent and identity of miRNA editing globally. Notably, the levels of seed editing of miR-376a-3p in hippocampal samples from TLE patients are significantly lower. Modelling this in human-induced pluripotent stem cell (iPSC)-derived neurons, we report that lowering the amount of the edited form of miR-376a-3p increases the expression of genes enriched in mitochondrial and metabolic pathways. These findings improve our understanding of the endogenous mechanisms and regulatory systems by which miRNAs shape the gene expression landscape in human epilepsy.

## Materials and methods

### Human brain tissue samples

Hippocampal tissue (cross-sectional study) was used in line with STROBE reporting guidelines.^24^ Briefly, hippocampal tissue was donated from patients who underwent surgical resection for treatment-resistant TLE and was provided by the Universitätsklinikum Frankfurt, Germany (*N* = 10), and Swedish Medical Centre, Seattle, USA (*N* = 1).^25^ The required sample size was estimated based on data from an analysis of epigenetic marks from a similar cohort.^25^ Informed consent was obtained according to the Declaration of Helsinki from all patients, and studies were approved by the Ethics Commission of the faculty of medicine, Goethe University Frankfurt (#4/09), Swedish Medical Center, Seattle, USA, and Beaumont Hospital Ethics (Medical Research) Committee (#5/18). Tissues used were removed as part of the normal clinical procedure, and no extra tissue was resected for research use. Hippocampal tissue from patients was graded for hippocampal sclerosis.^26^ Sclerotic and nonsclerotic samples were treated as a single group and compared to control (autopsy) hippocampus (*N* = 10) sourced from the NIH NeuroBioBank, USA. Samples were chosen to match the gender and age of the patients as closely as possible. Supplementary Table 1 contains the clinical and demographic details of the patients and controls.

### Western blotting

After extraction, proteins were separated by sodium dodecyl sulfate–polyacrylamide gel electrophoresis and then transferred to nitrocellulose membranes. The following primary antibodies were used: anti-ADAR1 (1:200; PA5-21369, ThermoFisher), anti-ADAR2 (1:200; 2248-1-AP, ProteinTech) and anti-beta-actin (1:5000; A5316, Sigma-Aldrich). Membranes were later incubated in secondary antibodies (1:10000; Abcam). Protein bands were detected using chemiluminescence (Merck) and captured using FujiFilm LAS-4000. Densitometry was performed using Image Studio Lite version 5.2.5.

### Immunohistochemistry

Hippocampal sections were post-fixed, permeabilized and blocked with bovine serum albumin (Sigma-Aldrich), then incubated overnight with anti-NeuN (1:400; MAB377, Sigma-Aldrich) and co-incubated with anti-ADAR antibodies (1:200 dilution). Sections were then washed and incubated with Alexa-conjugated secondary antibodies (1:400; Invitrogen). Sections were then stained to visualize nuclei using Hoechst (1:10000; Thermo Fisher Scientific) and coverslipped. Images were captured under a Nikon Eclipse 90i microscope with a Nikon DS-Ri1 camera. Image stitching of fields of view was performed using Nikon-NIS Element software, and immunofluorescence was analysed using Fiji software version 2.9.0. ^27^

### Cell-type-specific expression of ADAR transcripts

To verify the neuronal cell types expressing *ADAR* transcripts in the human brain (*ADAR* and *ADARB1*), we analysed single-nuclei RNA-sequencing data from the temporal cortex of TLE patients and controls.^28^ All plotting and analyses were done in R using code adapted from https://github.com/khodosevichlab/Epilepsy19. Filtered count matrices and cell subtype assignments were obtained as described.^28^ Joint graphing of single cells was performed using Conos version 1.5.0.^29^ Pseudobulk differential expression analysis was performed using DESeq2 version 1.40.1.^30^ Scripts used for these analyses are deposited to GitHub and available at https://github.com/Javizuma/ADAR_analysis.

### Detection of A-to-I modification and differential miRNA editing

Detection of miRNA editing was performed on small RNA-sequencing data generated from the hippocampus of TLE patients and controls.^23^ A total of eight and sixteen data sets from controls and patients, respectively, were downloaded from the European Genome-phenome Archive (EGA), under the accession number EGAS00001003922. Editing events in miRNAs and the analysis of differential editing between patients and controls were detected as previously described.^31^ Briefly, sequencing data were uploaded to ChimiRA webserver where adapters were trimmed and reads were mapped to miRbase version 21 hairpin sequence.^32^ The annotation of ADAR modifications and count-based evaluation of edited miRNAs was also performed using ChimiRA. Data were then subjected to an in-house bioinformatics pipeline generated from custom Python and bash scripts. Only miRNAs with at least 10 read counts carrying A-to-I modifications were considered for the identification of editing sites. Assuming the expected sequencing error rate of 0.01, a binomial cumulative distribution of *n*, *P* = 0.01 was applied to exclude modifications potentially from sequencing errors. MiRNAs that exhibited less than 10% of editing levels were filtered away. Then, an unpaired two-tailed *t*-test with Welch’s correction was used to identify the differences of A-to-I editing between controls and patients. The editing level was defined as the ratio of the read count of each non-reference modification to the total read count of the miRNA where A-to-I editing occurred within the mature miRNA. A Benjamini–Hochberg false-discovery rate (FDR) was applied and only editing sites that passed the FDR (*P* < 0.05) threshold were considered as differentially edited miRNA between controls and patients. All statistical calculations were performed in R environment and scripts used in this analysis were deposited to GitHub (https://github.com/NgocTNguyen/mirna-differential-editing-analysis). For Spearman's rank correlation coefficient, raw count matrices from the human and iPSC RNA-sequencing experiments were normalized using DESeq2’s median of ratios methods.^30^ Spearman’s correlation coefficient was then calculated between the normalized expression of all genes expressed in each condition; hippocampus of control individuals, hippocampus of individuals with temporal lobe epilepsy, iPSCs treated with a scrambled antimir, samples treated with antimirs targeting the edited form of miR-376a-3p (Anta-E) and the unedited form of miR-376a-3p (Anta-N). The mean normalized expression of the genes across the brain and iPSC data ignoring condition was calculated, and then Spearman’s correlation coefficient was calculated.

### In silico target analysis and iCLIP analysis

Prediction of genes targeted by the edited and non-edited miR-376a-3p was performed using miRDB,^33^ while the *in situ* targets were identified by interrogating data from individual-nucleotide resolution ultraviolet crosslinking and immunoprecipitation (iCLIP).^34^ Briefly, for miRDB, the biological targets were predicted based on the binding sites of the seed region. ‘Hsa-miR-376a-3p’ was used as an input for the non-edited form, while custom prediction was used for the edited form, where the edited site +6 within the mature sequence of miR-376a-3p 5’-AUCAU***G***GAGGAAAAUCCACGU-3’ was submitted as an input. Prediction scores above 50 were accepted. The *in situ* targets of the edited and non-edited miR-376a-3p were identified based on RNA targets cross-linked to argonaute 2 (AGO2) in the hippocampus from TLE patients, as reported.^34,35^ In brief, tissue samples were cross-linked by ultraviolet at 254 nm, and cross-linked protein/RNA was eluted after AGO2 immunoprecipitation. Sequencing, mapping and alignment to the human reference genome (GRCh38/hg38) allowed the detection of significant iCLIP peaks.^36^ MiRNA target interactions (MTIs) were employed to search for seed-complementary sites within the significant peaks, which was extended for the current study to include the edited and non-edited form of the mature miR-376a-3p sequence at site +6. Identified MTIs were intersected with predicted miRNA targets from TargetScanHuman version 8.0 to calculate the overlap of predicted and detected MTIs.^37^ Only genes with 7mer-A1, 7mer-m8 and 8mer binding sites were considered as the validated targets for the miR-376a-3p isomiR. The full iCLIP data can be obtained in Gene Expression Omnibus under the accession number GSE214317.

### Human iPSC-derived neurons

Human iPSC-derived neurons (line CRA_1302a) were provided by Roche and approved by the RCSI Research Ethics Committee (REC202302020). The cells were generated from blood cells obtained from a healthy female volunteer, 50 years of age. Briefly, plates were coated with 10 ug/ml LnBiolaminin-521 (BioLamina) in Dulbecco’s phosphate-buffered saline (Gibco) containing calcium and magnesium 1 day prior to use. Neural precursor cells were seeded at a density of 12 000 cells/cm^2^ in a 75T-flask pre-coated with 5 µg/ml Biolaminin-521 (BioLamina), grown in neuronal differentiation medium supplemented with sonic hedgehog (PeproTech), fibroblast growth factor 8 (PeproTech) and L-ascorbic acid 2 phosphate (Sigma-Aldrich) for 7 days at 37°C. After, cells were reseeded in well plates pre-coated with 5 µg/ml Biolaminin-521 and cultivated at a density of 45 000 cells/cm^2^ in neuronal differentiation medium supplemented with L-Ascorbic Acid 2-phosphate, brain-derived neurotrophic factor, dibutyrl adenosine 3’5’-cyclic monophosphate sodium salt, glial-cell-derived neurotrophic factor and 1% Penicillin–Streptomycin at 37°C with 5% CO_2_ for at least 31 days to ensure continued differentiation and maturation. Cortical neurons were then treated with 300 nM antimirs targeting the edited (5’-GATTTTCCTCCATGA-3’, Anta-E, Roche) or non-edited (5’-GATTTTCCTCTATGA-3’, Anta-N, Roche) forms of miR-376a-3p at the mature site +6, and scramble (Qiagen) at 37°C with 5% CO_2_ for 48 hours.

Cell viability was determined using a colorimetric assay (Reagent WST-1, Merck 5015944001). Briefly, 200 µl of media was collected 48 h after treatment and transferred to 96 well plates in triplicate and then 20 µl of WST-1 solution was added (1:10 final dilution). After incubation, the absorbance was measured at 450 nm using a spectral scanning reader (Thermo Scientific Varioskan Flash). Readings in Anta-E and Anta-N were normalized and expressed as a percentage of scrambled-treated cells.

iPSC-derived neurons differentiated for 8 weeks in vitro were selected for patch-clamp experiments. Whole-cell voltage-clamp recording was carried out with a Multiclamp 700 B amplifier (Molecular Devices, California, USA) which was interfaced by an A/D-converter (Digidata 1550B, Molecular Devices) to a PC running pClamp software (Version 11, Molecular Devices). The signals were low-pass filtered at 2 kHz and sampled at 10 kHz. All recordings were performed at 32°C in a bath solution containing (in mM): 135 NaCl, 3 KCl, 2 CaCl_2_, 1 MgCl_2_, 10 HEPES and 10 glucose (pH, 7.2; osmolality, 290–300 mmol/kg).

For recording spontaneous activity, neurons were loaded at 37°C with Cal-520 (AAT Bioquest, California, USA) for 45 min of incubation at 2 μM in culture media. After 45 min, cells were washed several times with dye-free (4-(2-hydroxyethyl)-1-piperazineethanesulfonic acid; HEPES)-buffered saline solution and transferred to an imaging chamber on a microscope (Zeiss Axio Examiner, Jena, Germany) equipped with a Zeiss 40 × water immersion objective. Zen Blue imaging software (Carl Zeiss) was used for hardware control and image acquisition, and analysis was performed using ImageJ. All imaging experiments were performed at 32°C in a solution with the composition (in mM): 135 NaCl, 3 KCl, 2 CaCl_2_, 1 MgCl_2_, 10 HEPES and 10 glucose (pH, 7.2; osmolality, 290–300 mmol/kg). Images were acquired at 2 Hz. For mitochondrial membrane potential imaging, cells were incubated with tetramethylrhodamine (TMRM; 100 nM),^38^ along with Cal-520 in culture medium for 45 min at 37°C in the same conditions used for Cal-520 imaging except that Cy 3.5 filter set was used.

### RNA isolation and RT-qPCR

Total RNA was isolated using TRIzol (Invitrogen); 250 ng of total RNA was used to generate cDNA using the MultiScribe Reverse Transcriptase kit (Thermo Fisher Scientific). Primers used for the reverse transcription and quantitative PCR were obtained from stem-loop miRNA TaqMan assay (Applied Biosystems). A custom miRNA TaqMan assay for the edited form of miR-376a-3p (5’-AUCAUGGAGGAAAAUCCACGU-3’) was made with Applied Biosystem, while the non-edited version can be found in the supplier reference number under #000565. Quantitative PCR of miRNA expression level was performed in QuantStudio 12k Flex machine. MiRNA levels were normalized to *RNU19*. The relative fold change in miRNA expressions was calculated based on the comparative threshold value of 2^−ΔΔCT^.

### Sanger sequencing

Sanger sequencing was performed by GENEWIZ from Azenta Life Sciences to validate the occurrence of editing at site +6 for miR-376a-3p in the human hippocampal tissues. Briefly, 500 ng of total RNA was treated with DNase 1, and cDNA was generated using the Superscript III Reverse Transcriptase (Invitrogen) and amplified with random hexamer primer based on the manufacturer’s protocol. The primer for miR-376a-3p used in Sanger sequencing was generated as previously described.^39^ EditR was later used in the chromatogram to perform base calling at site +6 of miR-376a-3p.^40^

### RNA-Sequencing and pathway enrichment analysis

Library preparations and RNA-sequencing were performed by Novogene Europe to analyse the differential gene expression (DEG) after treatment of neurons with Anta-N and Anta-E in comparison with cells treated with scramble (*n* = 8 for each). Libraries were sequenced by an Illumina NovaSeq 6000, and data were subjected to paired-end sequencing, with a read length of 150 bp to a depth of 20 million reads. Low-quality and adapter-containing reads were removed, and clean reads were mapped to the GRCh38/hg38 reference genome using Hisat2 version 2.0.5. Reads mapped to each gene were counted using featureCounts version 1.5.0-p3, and differential expression was performed using DESeq2. Only genes with log_2_ fold change *n* > 0 or *n* < 0 together with adjusted *P* values <0.05 were considered differentially expressed. Pathway enrichment analysis was performed using ShinyGO version 0.75 on DEGs that were upregulated after Anta-E treatment. ^41^ For the pathway enrichment analyses, Gene Ontology (GO) Biological Process, REACTOME, and Kyoto Encylopedia of Genes and Genomes (KEGG) were utilized. Only the top 10 pathways with FDR values <0.05 were considered significantly enriched. RNA-sequencing data were deposited into Gene Expression Omnibus and available under the accession number of GSE211696.

### Statistical analysis

Statistical analyses were conducted using GraphPad Prism version 9 unless stated otherwise. Unless otherwise described, data were presented as mean ± SEM and analysed for ordinary two-tailed *t*-test or one-way ANOVA with Bonferroni corrections when appropriate. Threshold values of *P* < 0.05 were considered statistically significant, and asterisks used in this study were denoted as *P < 0.05, **P < 0.01, ***P < 0.001 and ****P < 0.0001.

## Results

### Hippocampal ADAR levels in TLE patients

**Figure 1:**
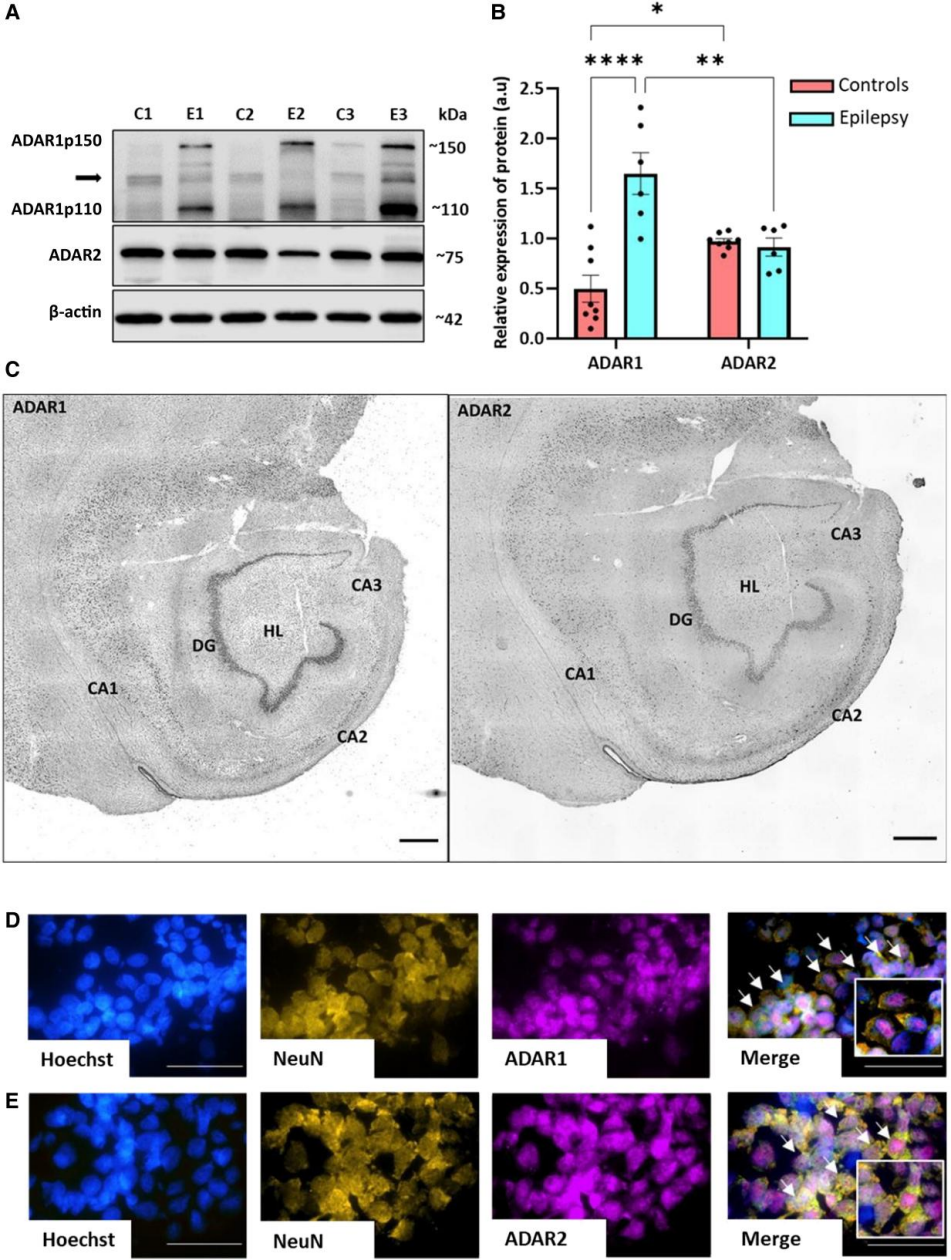
**Adenosine deaminase acting on RNA (ADAR) proteins in hippocampus from temporal lobe epilepsy patients.** (**A**) Representative western blot showing ADAR1 (∼150 kDa, ADAR1p150; ∼110 kDa, ADAR1p110) and ADAR2 (∼75 KDa) protein levels in hippocampal tissues of autopsy controls (controls) and drug-resistant temporal lobe epilepsy (TLE) patients (epilepsy). β-actin is shown as a guide to loading and was used for normalization. Symbol (−>) Indicates unspecific bands. Uncropped blots are included in Supplementary Fig. 1. (**B**) ADAR1 and ADAR2 protein levels in the hippocampus of TLE patients when compared to controls. (Mean ± SEM, *n* = 8 controls, 6 epilepsy, One-way ANOVA with Bonferroni correction, *P < 0.05, **P < 0.01, and ****P < 0.0001). (**C**) Representative staining of ADAR1 and ADAR2 in different sub-fields of the hippocampus from a TLE patient (scale bar 1000 µm, total magnification of 40x. CA: Cornu Ammonis, DG: Dentate gyrus, HL: Hilus). Note, both ADARs present in each sub-field of the hippocampus. (**D, E**) Representative immunostaining showing **D** ADAR1 or **E** ADAR2 overlaps with neuronal nuclei marker (NeuN) in the hippocampus of a TLE patient. Hoechst dye was used as a nuclear stain for DNA (scale bar 50 µm, total magnification of 1000 × with oil immersion). A arrowheads indicate the presence of overlapping signals, and the magnified version is shown in the box of each panel.

Since ADARs mediate miRNA editing in the brain, we first sought to establish the presence of the major ADAR isoforms in the surgically-obtained hippocampus of TLE patients and autopsy controls. Western blotting using specific antisera detected ADAR1 (ADAR1p110 and ADAR1p150) and ADAR2 at their expected molecular weights in the hippocampus (Fig. 1A and Supplementary Fig. 1). Levels of ADAR1 (ADAR1p110 and ADAR1p150 together) were about three times higher in the hippocampus of TLE patients, whereas ADAR2 protein levels were comparable to controls (Fig. 1B and Supplementary Fig. 1). Post-mortem interval did not have a significant effect on ADAR protein levels (Supplementary Fig. 2).

These findings were supported by immunohistochemistry. ADAR1 and ADAR2 immunoreactivity was prominent within the major neuronal cell layers in controls and patient sections (Fig. 1C and Supplementary Fig. 3). Counterstaining ADAR1 or ADAR2-labelled sections with antibodies against the neuronal marker NeuN confirmed the cellular identity of most ADAR staining as neuronal (Fig. 1D and E). Non-neuronal cells including astrocytes displayed weak or no immunoreactivity for ADAR1 and ADAR2 (Supplementary Fig. 4). To further corroborate the immunohistochemical findings, we interrogated single-cell tissue expression data obtained from human controls and TLE patients.^28^ This analysis showed that both *ADAR* transcripts are expressed in neurons in the human temporal cortex. While the levels of *ADAR*s were comparable across many neuronal subtypes, two specific subtypes of inhibitory neurons exhibited lower levels of *ADAR2* (Supplementary Fig. 5). Overall, these data indicate the main miRNA editing enzymes are neuronally enriched in TLE patients.

### Genome-wide survey of miRNA editing in human TLE

**Figure 2:**
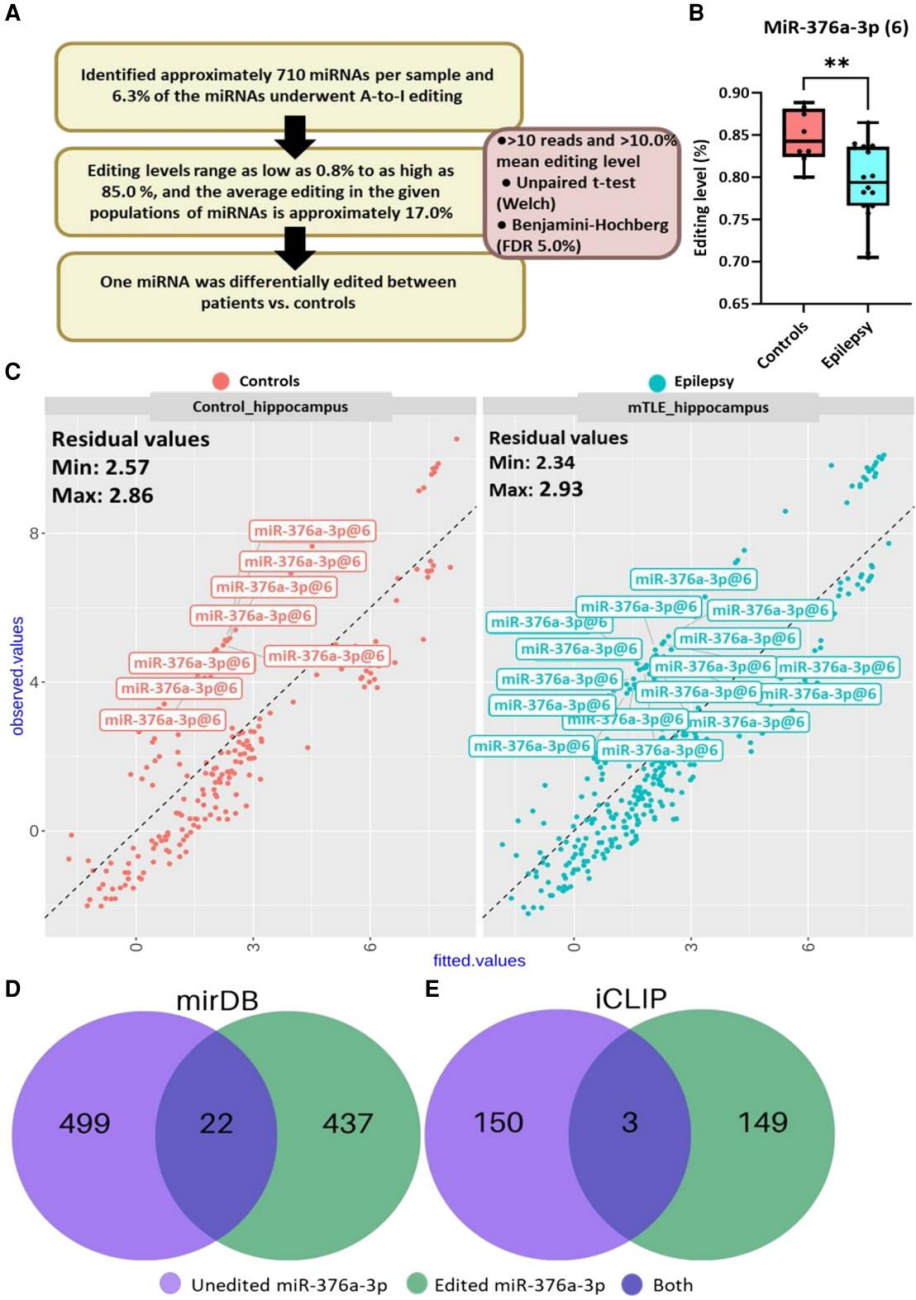
**Differential editing of miRNAs in human temporal lobe epilepsy.** (**A**) Schematic of the editing landscape in human hippocampus and the criteria for detecting differential editing sites in mature microRNAs (miRNAs) between controls and mesial temporal lobe epilepsy (mTLE/TLE) patients (*n* = 8 controls, 16 epilepsy). (**B**) Graph showing the editing levels of miR-376a-3p (site +6) in the hippocampus of TLE patients and controls. (Mean ± SEM, *n* = 8 controls, 16 epilepsy, Welch’s **T** test with Benjamini–Hochberg correct fisher exact test, **P < 0.05). (**C**) Correlation of the edited form for miR-376a-3p (site +6) against the total abundance of the non-edited form. Data points distributed above and away from the coefficient fitted line indicated miR-376a-3p editing is greater than expected based on the total abundance. (**D**) Predicted change observed in mRNA targets between non-edited and edited miR-376a-3p using miRDB and (**E**) individual-nucleotide resolution ultraviolet crosslinking and immunoprecipitation (iCLIP) database.^34^ Less than 5.0% of the targets are shared between the isomiRs.

To characterize the occurrence and extent of miRNA editing in human TLE, we extracted small RNA-sequencing data from a set of 24 hippocampi from controls and TLE patients.^23^ Analysis of miRNA editing was performed as described.^31,32^ Briefly, small RNA-sequencing reads were uploaded to ChimiRA, annotation of the A-to-I changes was carried out using the ChimiRA algorithm, and further statistical analyses were performed to identify overrepresented A-to-I modifications within the samples (Fig. 2A).

The interrogated miRNA data contained approximately 710 miRNAs per sample. Among the detected miRNAs were brain-enriched miRNAs including miR-9-5p, miR-128-3p and miR-181a-5p as well as lower expressed miRNAs previously associated with human TLE such as miR-134-5p and miR-146a-5p.^15^ We also detected multiple miRNAs that were previously reported to be edited such as miR-200b,^42^ miR-589,^43^ miR-376a^44^ and miR-376b.^45^ (Supplementary Fig. 6).

A-to-I modifications were detected in 6.3% of the miRNAs (45 miRNAs) across TLE and control samples (Supplementary Table 2). The editing level ranged from as low as 0.8% to as high as 85.0% (Fig. 2A). The editing ratio per base site was similar between those which fell within the seed (26) and outside the seed (19) among the edited miRNAs. Edited miRNAs were mapped to chromosomal positions, which showed broad distribution with the highest numbers located on chromosomes 1 (8) and 14 (11) (Supplementary Fig. 6). We then searched for motifs within the seed region where miRNA editing was most common. This revealed the highest number of editing events occurred for adenosine-containing 5’-UAG-3’ sequences, consistent with known preferences for ADAR-mediated editing (Supplementary Fig. 7).

### Differential seed editing of miR-376a-3p in human TLE

Low-level RNA editing, for example below 10%, may not be biologically relevant for validation studies.^39,46,47^ Accordingly, we filtered out miRNAs that exhibited editing below 10% (that is, less than 1/10 copies of the miRNA exhibiting editing). We next compared the relative levels of the remaining edited miRNAs between TLE patients and controls. Overall, the mean amount of editing per miRNA was very similar between patients and controls, indicating limited differential editing in human TLE. Only a single edited miRNA, miR-376a-3p, passed correction for multiple comparisons (*P* < 0.05) and was found to be differentially edited in TLE patients compared to controls (Fig. 2B).

**Figure 3:**
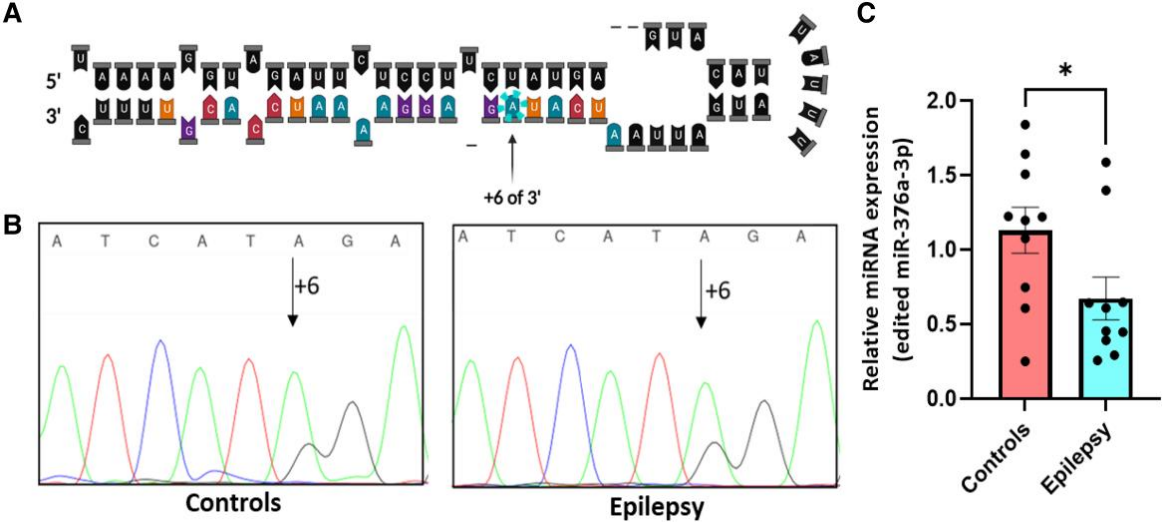
**Stem-loop structure and the expression of miR-376a-3p in human temporal lobe epilepsy.** (**A**) Edited adenosine identified by a blue circle is located at position +6 on the 3p arm of the stem-loop sequence of pri-miR-376a. Figure created using Biorender.com. (**B**) Chromatogram from Sanger sequencing showing the edited site of miR-376a-3p (G peak, black) in the hippocampus from controls (left) and patients (right). (**C**) Graph illustrates the relative expression levels of the edited miR-376a-3p in the hippocampus of both control subjects and those with temporal lobe epilepsy (TLE) (Mean ± SEM, *n* = 10, Ordinary two-tailed **T** test, *P < 0.05). Expression level were calculated as 2^−ΔΔCT^, and expression values were normalized to RNU19 mRNA.

Mature miR-376a-3p is a 21 nucleotide miRNA that is derived from a locus on the long arm of chromosome 14 (14q32), is part of an imprinted miRNA cluster,^48,49^ and a moderately expressed, brain-enriched miRNA.^50^ This indicates high potential biological relevance. The mean editing level observed in controls was 85.0% and this was significantly lower, 79.0% in TLE patient samples (Fig. 2B). The site of editing was within the seed region, at nucleotide position 6 from the 5’ end of the miRNA [miR-376-3p(+6) (and see Fig. 3A)]. To ensure that the lower editing level was not simply due to lower abundance, we performed a Pearson’s correlation test between the abundance of the edited form against the overall abundance of miR-376a-3p (Fig. 2C). The residual values of miR-376a-3p (+6) abundance among the patients ranged from 2.34 (minimum) to 2.93 (maximum), which were distant from the fitted line (a zero value for a perfect fit model; Fig. 2C). This indicates that the observed reductions of the edited form miR-376a-3p (+6) are not influenced by any potential changes in the overall abundance of miR-376a-3p in the patients.

### Editing of miR-376a-3p shifts the target pool

To explore potential downstream functional effects of altered levels of A-to-I editing, we combined *in silico* target prediction using miRDB,^51^ with experimentally validated targets within the RNA-induced silencing complex.^34^ The latter involved RNA–protein crosslinking of immunoprecipitated AGO2 (iCLIP) from a set of resected hippocampi from TLE patients, followed by sequencing and alignment of miRNA-mRNA target pairs. This identifies high-confidence mRNAs under active miRNA control. The miRDB algorithm predicted over 400 targets of both non-edited and edited forms of miR-376a-3p. Importantly, miRDB identified only 22 targets that overlapped between the non-edited and edited miR-376a-3p(+6) (Fig. 2D). Similar results were found when the iCLIP dataset was interrogated. Around 150 mRNAs within the AGO2 complex in human TLE hippocampal samples were targets of non-edited and edited forms of miR-376a-3p, with only three in common (Fig. 2E). Thus, only a few percent of the predicted or *in situ* AGO2-loaded targets overlap between non-edited and edited forms of miR-376a-3p. These findings also suggest that the functional pool of miR-376a-3p targets is approximately one-third the potential number (i.e. predicted by miRDB). Taken together, these results indicate that a single base change or reduced editing at the seed region of position 6 in miR-376a-3p can result in significant redirection of target selection.

We next sought to validate these findings using samples from an independent cohort of control and TLE patient samples. We first used Sanger sequencing to verify that miR-376a-3p is edited at site +6 in both control and TLE hippocampus. Based on Sanger sequencing, a G peak was visible at site +6 of miR-376a-3p in samples consistent with this site being targeted for ADAR editing (Fig. 3B). To quantify this difference, we employed a custom-made TaqMan miRNA assay that utilizes target specific stem-loop primers to measure the relative expression of the edited transcript of miR-376a-3p. The stem-loop structure of miR-376a and the edited position 6 of the 3p arm are depicted in Fig. 3A. The TaqMan assay revealed that levels of the edited transcript of miR-376a-3p (+6) were significantly lower in TLE patients compared to controls (Fig. 3C), while the non-edited transcripts remain comparable between groups (Supplementary Fig. 8).

### Modulation of the edited form of miR-376a-3p in human iPSC-derived neurons

**Figure 4:**
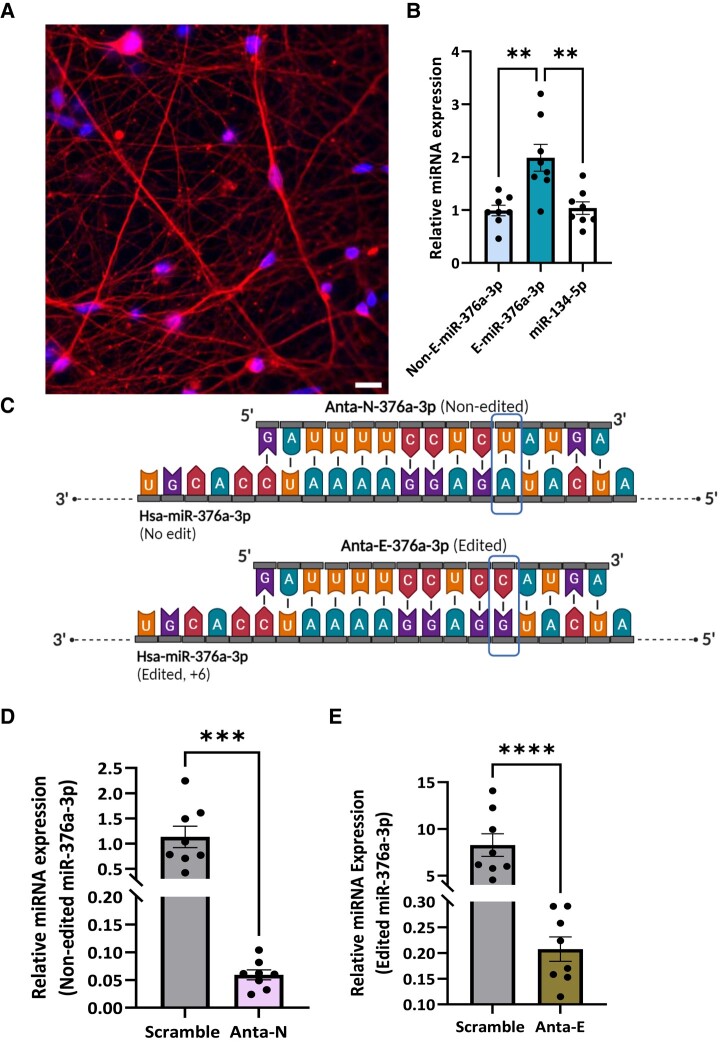
**miR-376a-3p isomiRs in human-induced pluripotent stem cell (iPSC)-derived neurons.** (**A**) Representative photomicrograph showing iPSC-derived neurons exhibit typical neuronal morphology and express beta-III-tubulin (TUJ1, red). Hoechst dye (blue) was used as a nuclear stain for DNA (scale bar 20 µm). (**B**) Relative expression of the endogenous miR-376a-3p isomiRs in neurons derived from iPSCs. Each data point corresponds to the miRNA's relative expression in each individual sample. (Mean ± SEM, *n* = 8, One-way ANOVA with Tukey corrections, **P < 0.01). Expression level were calculated as 2^−ΔΔCT^, and expression values were normalized to RNU19. (**C**) Antisense oligonucleotides for Antimir-Non-edited (Anta-N) and Antimir-Edited (Anta-E) used to silence non-edited (top) and edited (bottom) miR-376a-3p, respectively. Figure created using Biorender.com. (**D, E**) Graphs show reductions of endogenous **D** non-edited and **E** edited miR-376a-3p after treatment of neurons with Anta-N and Anta-E, respectively. Scramble (scrm) was used as a control. Each data point corresponds to the miRNA's relative expression in each individual sample after treated with either scrm, Anta-N, or Anta-E. (Mean ± SEM, n = 8 per treatment group, Ordinary two-tailed **T** test, ***P < 0.001, ****P < 0.0001).

We next used human iPSC-derived neurons to explore if changes to miRNA editing affect the gene expression landscape. After 30 days of differentiation, cells displayed typical markers of neuronal identity and maturation (Fig. 4A and Supplementary Fig. 9). This included transcripts expressed by mature excitatory and inhibitory neurons, morphology of likely inhibitory (bipolar) and excitatory (pyramidal) neurons, and miniature excitatory and inhibitory post-synaptic currents (Supplementary Fig. 9).

Next, the same TaqMan assays were used to establish the presence of non-edited and edited forms of miR-376a-3p. We also measured levels of miR-134-5p, an epilepsy-associated,^15^ neuronally enriched miRNA expressed at a low-to-moderate level in the human brain.^17,50^ RNA from the iPSC-derived neurons contained detectable levels of both non-edited and edited miR-376a-3p (Fig. 4B). Levels of the non-edited form of miR-376a-3p were similar to levels of miR-134-5p. The relative expression of the edited form of miR-376a-3p(+6) was higher than its non-edited counterpart (Fig. 4B). Thus, these neurons express clinically relevant levels of edited and non-edited miR-376a-3p.

We next designed antimirs against the non-edited (Anta-N) and edited forms (Anta-E) of miR-376a-3p (Fig. 4C). Taqman assays confirmed that treatment (48 hours) of iPSC-derived neurons with either Anta-N or Anta-E reduced levels of the two forms of miR-376a-3p (Fig. 4D and E). Cell viability remained high in cells exposed to either antimir (Supplementary Fig. 10).

### Reducing miR-376a-3p isomiRs results in distinct gene expression profiles in human neurons

**Figure 5:**
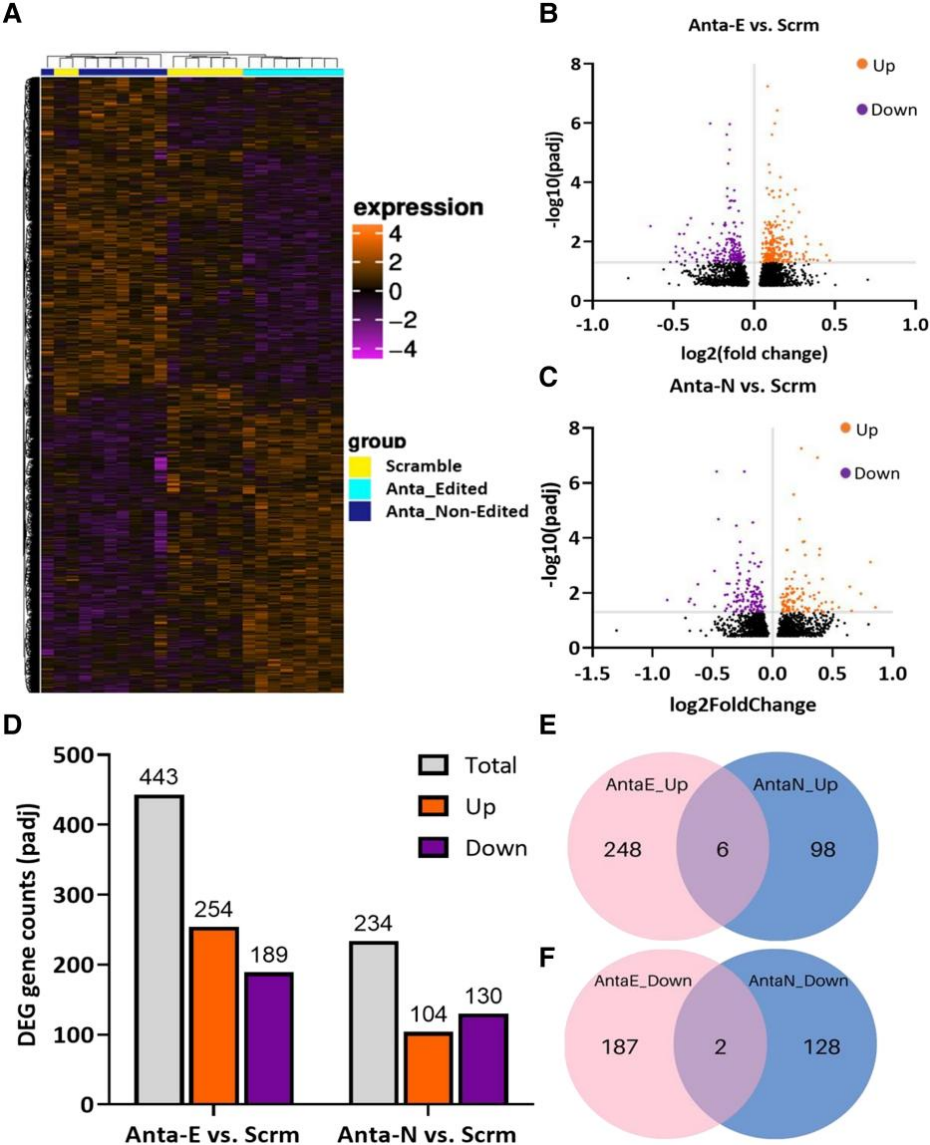
**Effect of altered miR-376a-3p editing on gene expression in human-induced pluripotent stem cell (iPSC)-derived neurons.** (**A**) Heatmap showing the clustering of groups based on the differentially expressed genes (DEGs) log_10_ (FPKM + 1) value. Increase (orange) or decrease (magenta) expression is represented on the scale. (**B, C**) Volcano plot of DEGs after treatment with **B** Antimir-Edited (Anta-E) or **C** Antimir-Non-edited (Anta-N) when compared to scrambled [scrm (*n* = 8 per group)]. E, edited. The orange scatters indicate upregulated DEGs, magenta scatters indicate downregulated DEGs, and black scatters indicate DEGs that failed to pass the multiple testing analysis (cut-off value at -log_10_P_adj_ <0.05 or absolute value > 1.3). Log_2_Fold change represents the difference in gene expression between Anta-E versus scramble. (**D**) Each bar plot displays the combined number of DEGs, with both upregulated and downregulated, as determined by the volcano plots in Figure (**B, C**). (*n* = 8 per treatment group). (**E, F**) Venn diagrams showing the number of **E** upregulated or **F** downregulated DEGs and overlap between neurons treated with Anta-E and Anta-N.

We next explored the effect of lowering levels of either the non-edited or edited form of miR-376a-3p in iPSC-derived neurons, on gene expression. Cells were treated as above and then mRNA was sequenced. Reducing levels of either form of miR-376a-3p resulted in distinctive gene expression profiles (Fig. 5A and Supplementary Fig. 1^1^). Differentially expressed genes (DEGs) were identified using an adjusted threshold value of Padj < 0.05 and log2 fold change. Inhibition of the edited form of miR-376a-3p using Anta-E resulted in a total of 443 DEGs (254 upregulated; 189 downregulated; Fig. 5B), whereas inhibition of the non-edited form resulted in 234 DEGs (104 upregulated; 130 downregulated; Fig. 5C), compared to scramble-treated cells (Fig. 5D). Additional DEGs, comparing between cells treated with Anta-E or Anta-N versus scramble, are depicted in Supplementary Fig. 1^2^. We next compared the overlapping DEGs between Anta-E and Anta-N when compared to scramble. A Venn analysis found fewer than 6.0% of the DEGs overlapped between these groups (Fig. 5E and F). This suggests inhibiting either the non-edited or edited form of miR-376a-3p produces divergent changes in gene expression, consistent with findings in human TLE samples, indicating that the edited and non-edited miR-376a-3p regulate largely distinct transcripts.

To gauge whether these gene expression profiles were clinically relevant, we performed a correlation analysis between the RNA-sequencing data from the iPSC-derived neurons to the original human hippocampus RNA-sequencing data.^23^ This showed a significant correlation of the expressed genes between the two samples (Supplementary Fig. 1^3^). Thus, the *in vitro* gene expression profile is relevant to human TLE.

### Enhanced metabolic pathways upon inhibition of the edited form of miR-376a-3p

**Figure 6:**
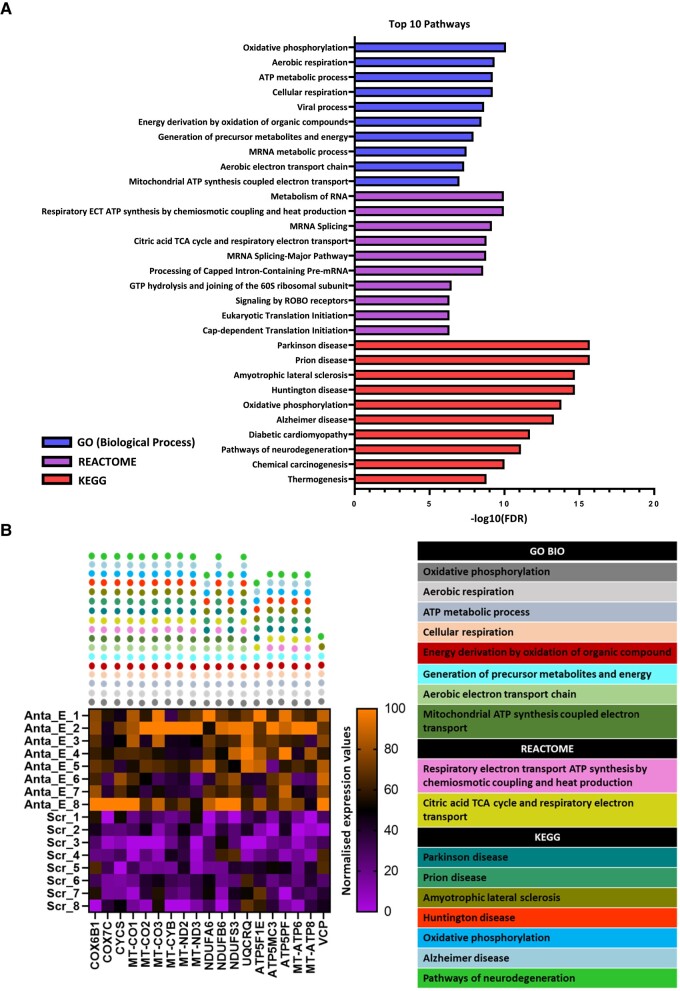
**Pathways affected by inhibition of edited miR-376a-3p in human neurons.** (**A**) Pathway enrichment analysis of differentially expressed genes (DEGs) for Antimir-Edited (Anta-E)-treated neurons compared to scrambled (scr), using Gene Ontology (GO) category of biological process, REACTOME and Kyoto Encyclopedia of Genes and Genomes (KEGG). Top 10 significantly enriched pathways of each category were selected based on the false-discovery rate (FDR, *P* < 0.05). (**B**) Heatmap showing the normalized expression values of the filtered upregulated DEGs between Anta-E versus scramble-treated neurons. Pathways are colour-coded, and the coloured circle on each gene indicates the specific enriched pathway associated with the gene (from *n* = 8 per group).

Finally, we explored the pathways enriched among upregulated DEGs following the inhibition of the edited form of miR-376a-3p in iPSC-derived human neurons. Pathway enrichment analysis was performed on the upregulated DEGs from Anta-E-treated neurons compared to scrambled using Gene Ontology (GO) for biological processes, REACTOME and Kyoto Encyclopedia of Genes and Genomes (KEGG) (Fig. 6A and Supplementary Table 3). Only pathways that passed the FDR <0.05 threshold were deemed to be statistically enriched.

GO analysis revealed upregulated DEGs were primarily enriched in metabolic pathways, including mitochondria-related activities such as oxidative phosphorylation, ATP metabolic process, cellular respiration and generation of precursor metabolites and energy. Similarly, REACTOME analysis found enriched metabolic and mitochondria-related pathways, including terms such as respiratory electron transport chain synthesis and citric acid-tricarboxylic acid and respiratory electron transport (Fig. 6A). KEGG analysis identified enrichment related to neurological and neurodegenerative diseases such as Parkinson disease, Amyotrophic lateral sclerosis and Alzheimer disease, as well as oxidative phosphorylation (Fig. 6A). Thus, reduced levels of the edited form of miR-376a-3p appear to alter energy metabolism and are associated with neurodegenerative phenotypes.

We explored in more detail the identities of the genes whose transcripts were upregulated upon inhibition of the edited form of miR-376a-3p and among the enriched pathways. This identified 19 genes in eight or more pathways related to mitochondrial functions (Fig. 6B). This was a mix of mitochondrial- and nuclear-encoded genes, including cytochrome C oxidase subunits 6B1 and 7C (*COX6B1, COX7C*), cytochrome C, somatic (*CYCS*), mitochondrially encoded cytochrome C oxidase 1, 2 and 3 (*MT-CO1*, *MT-CO2* and *MT-CO3*), mitochondrially encoded cytochrome B (*MT-CYB*), mitochondrially encoded NADH:Ubiquinone oxidoreductase core subunits 2 and 3 (*MT-ND2*, *MT-ND3*), NADH:Ubiquinone oxidoreductase subunit A6 and B6 (*NDUFA6*, *NDUFB6*), ubiquinol-cytochrome C reductase complex III subunit VII (*UQCRQ*), ATP synthase F1 subunit epsilon (*ATP5FIE*), ATP synthase membrane subunit C locus 3 (*ATP5MC3*), ATP synthase peripheral stalk subunit F6 (*ATP5PF*) and mitochondrially encoded ATP synthase membrane subunits 6 and 8 (*MT-ATP6, MT-ATP8*) (Fig. 6B). Thus, a major effect of reducing the edited miR-376a-3p is the upregulation of mitochondrial genes in neurons. Any dysregulation of the level of miR-376a-3p editing in the brain could lead to metabolic reprogramming that in turn may affect the function and resilience of neurons in human TLE.

**Figure 7:**
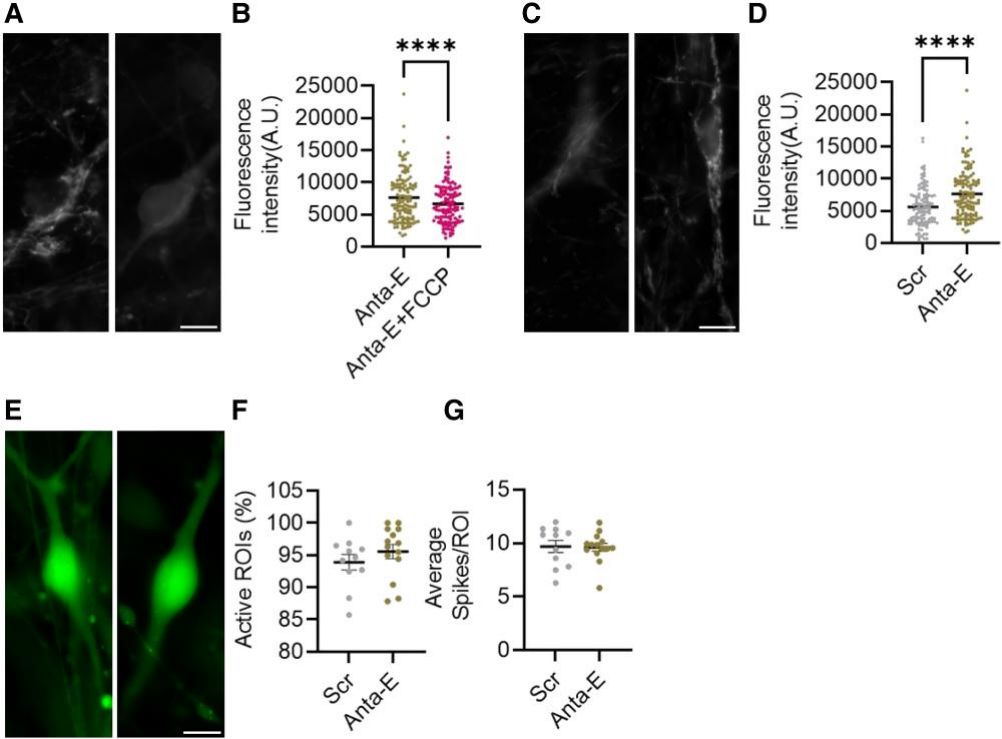
**Inhibiting the edited form of miR-376a-3p increases mitochondrial membrane potential in human neurons.** (**A**) Representative images of human neurons loaded with 100 nM tetramethylrhodamine (TMRM) before (left) and after (right) Carbonyl cyanide-*P*-trifluoromethoxyphenylhydrazone (FCCP, 1 µM) application. (**B**) Disruption of mitochondrial membrane potential upon FCCP application (Wilcoxon matched-pairs signed rank test, *P* < 0.0001, *n* = 119 imaged cells). A.U., arbitrary units. (**C**) Representative images of human neurons treated with scrambled (Scr, left) or Antimir-Edited (Anta-E, right) loaded with 100 nM TMRM. (**D**) TMRM fluorescence intensity in Scr- or Ant-E-treated neurons (Mann–Whitney U test, *P* < 0.0001, N = 100 or 119 in Scr or Anta-E, respectively). (**E**) Representative images of neurons treated with Scr (left) or Anta-E (right) loaded with the calcium indicator Cal-520. (**F**) Percentage of active neurons after treatment with Scr or Anta-E. ROI, region of interest. (**G**) Average number of spikes per neuron in Scr or Anta-E-treated neurons (unpaired *t*-test, *P* = 0.3194, *N* = 1232 or 2033 in Scr and Anta-E, respectively). (**G**) Average number of spikes per neuron in Scr or Anta-E-treated neurons (unpaired *t*-test, *P* = 0.9034, *N* = 1232 or 2033 in Scr and Anta-E, respectively).

We hypothesized that the altered gene expression resulting from inhibition of the edited form of miR-376a-3p may result in functional changes to mitochondrial membrane potential. To test this idea, we analysed mitochondrial membrane potential using tetramethylrhodamine and performed calcium imaging in iPSC-derived neurons treated with Anta-E to lower the levels of the edited form of miR-376a-3p (Fig. 7). Treatment of neurons with a mitochondrial uncoupling agent produced the expected drop in tetramethylrhodamine signal (Fig. 7A and B). Analysis of tetramethylrhodamine measurements in Anta-E-treated neurons found a higher signal compared to scrambled-treated neurons consistent with a more negative resting potential (Fig. 7C and D). In contrast, Anta-E did not affect spontaneous activity in neurons as measured by calcium imaging (Fig. 7E-G).

## Discussion

Although miRNAs are implicated as important regulators of the gene expression landscape in human TLE and other forms of treatment-resistant epilepsy, emphasis to date has been on changes to the abundance of miRNAs. This has been supported by experimental studies showing that evoked and spontaneous seizures can be modified by altering miRNA levels in rodents.^15,19^ Here, we explored changes to the miRNA sequence, via editing, a process mediated by ADARs.^16,20,21^ RNA editing is a potent molecular tool used by cells to diversify the protein-coding transcriptome and generate changes to receptors and signalling pathways that can alter brain excitability in health and disease, including epilepsy.^14^ Likewise, editing of miRNAs can profoundly change their effects by altering their biogenesis or redirecting targeting.^20,52^

The present study mined RNA-sequencing data on the miRNAome,^23^ to evaluate the miRNA editing landscape in human TLE. Our findings confirm other studies that a specific number of miRNAs are normally edited in the human brain, which varies in a miRNA-dependent manner.^21,22,53^ The selection of miRNAs for editing depends on specific motifs recognized by ADAR enzymes,^52^ although the factors affecting the extent of editing of a given miRNA remain incompletely understood. Although editing can affect miRNA maturation, including by altering the sequence of the hairpin precursors,^52^ we focused on changes to the mature sequence, finding editing both within and outside the seed. This included multiple examples of miRNAs previously reported as substrates for ADAR-mediated editing, including the miR-376 cluster of miRNAs.^21,54^ Among these, we detected several heavily edited miRNAs, including miR-376a-3p.^21,54^ Thus, the current findings in the hippocampus of TLE patients are reflective of previous findings on miRNA editing in the human brain. A key finding here was the majority of miRNA editing in the hippocampus was similar between TLE patients and controls. This was unexpected, given the extensive dysregulation of miRNA levels in the hippocampus of TLE patients,^23,55^ and differences in editing of protein-coding genes in experimental TLE.^14^ Moreover, and despite being neuron-enriched and therefore potentially depleted due to the neuron loss common in such material, we found ADAR1 levels were elevated in the resected hippocampus from TLE patients. While the editing and ADAR analyses were performed using separate sample cohorts, the findings indicate distinct coding and noncoding editing landscapes in TLE. Thus, miRNA editing may be less sensitive or more resilient to the pathophysiology of epilepsy when compared to protein-coding transcripts.^14^ The present study did not explore the mechanism causing the elevated ADAR1 levels in the hippocampus of TLE patients. Notably, the production of ADAR1 and RNA editing is sensitive to inflammatory signalling,^56^ which is a common finding in the hippocampus of TLE patients.^57^

A-to-I editing has been reported in multiple regions of precursor and mature miRNAs. The editing of miRNAs within the seed region can substantially change the target pool.^21,39,54^ The *in vivo* brain functions of the non-edited form of miR-376a-3p are poorly understood and there are few examples of changes to miRNA editing linked to the pathogenesis of brain-related diseases.^44,58^ The present study included validation of the high editing level and differences in miR-376a-3p editing between controls and TLE patients,^23^ in an independent set of hippocampal samples. Thus, despite being a small change in editing, the finding is reproducible. In the present study, we used both predictive tools and cross-linked targets within the miRNA-induced silencing complex to confirm that the edited and non-edited forms of miR-376a-3p have few targets in common. That is, the site +6 seed-editing event causes a major switch in the target pool of this miRNA. The impacted pathways included biological processes with high relevance to epilepsy and include both loss- and gain-of-targets. This was supported by functional studies that explored the effect of reduced levels of the edited form of miR-376a-3p in human iPSC-derived neurons. Although the repressive effect of individual miRNAs on targets is typically small,^16^ we found widespread and significant changes to hundreds of transcripts when either the edited or non-edited form of miR-376a-3p was downregulated. This is consistent with miR-376a-3p controlling an extensive network of genes. Lowered levels of edited miR-376a-3p resulted in a highly specific increase in transcripts related to mitochondrial function and metabolism in human neurons, including multiple components of the mitochondrial respiratory chain. Notably, select changes to mitochondrial complex proteins has been reported in tissue samples from TLE patients.^59^ Metabolic reprogramming is increasingly linked to disease mechanisms, including control of inflammatory responses,^60^ and changes to metabolic pathways are implicated in human TLE,^61^ and well as mitochondrial epilepsy.^62^ Mitochondrial function is also critical for intracellular calcium homeostasis.^63^ Functional studies here began to parse these possibilities, showing a reduction in miR-376a-3p editing leads to a slightly more negative mitochondrial membrane potential but not a change in calcium signalling. Enhanced mitochondrial and metabolic function may increase resilience to the energy, oxidative stress or excitatory drive demands in epileptogenic tissue.^38^ However, the shut-down of energy-intensive pathways is also associated with endogenous mechanisms of neuroprotection including states of hibernation.^64^

Both up- and downregulated genes were detected upon reduction of the edited form of miR-376a-3p. This likely derives from de-repression of direct targets as well as indirect effects and changes secondary to reduced targeting of transcriptional repressors. Notably, only upregulated transcripts after reduction of the edited form of miR-376a-3p were associated with statistically enriched pathways. Among other pathways altered were some associated with neurodegenerative disease. Taken together, a shift in the editing of this miRNA likely represents a form of molecular repurposing that is highly efficient and expansive in its effects. Given the anti-seizure effects reported for several targeted miRNAs in preclinical models,^15,19^ future studies could pursue whether the miRNA editing-associated gene expression changes underpin functionally significant responses that attenuate or promote hyperexcitability. It may be possible to change miRNA editing levels *in vivo* and evaluate impacts on seizures or co-morbidities in rodent models or *in vitro* in human brain slices obtained following surgical resection.^65^

There are potential limitations to the present study. Editing of protein-coding transcripts shows distinct cell-type-specific patterns in the human brain.^66^ Here we used bulk tissue to identify miRNA editing which masks potential cell-type-specific editing events. For our functional studies, we detected an appropriate ratio of edited to non-edited miR-376a-3p in iPSC-derived neurons but it is unlikely the reduction in levels of edited and non-edited forms of miR-376a-3p we achieved using antimirs matches exactly the different levels observed in human brain. Moreover, iPSC-derived neurons represent imperfect models of the adult brain.^67^ Detecting single nucleotide differences can be unreliable, and additional quantitation methods should be considered in future studies.^68^ Larger sample cohorts may enable studies comparing editing differences between pathological grades and explore whether differences exist beyond the hippocampus in TLE or in other forms of focal, treatment-resistant epilepsy. Finally, the present study does not establish the mechanism for the specific change in miRNA editing level.

In conclusion, the present study reveals that miRNA editing is preserved and largely unaltered in human TLE. Only editing of miR-376a-3p exhibited statistically significant changes in TLE tissue but this had still a major effect on the target pool. The findings have implications for mechanisms regulating miRNA function in epilepsy and may lead to novel approaches to targeting miRNA for seizure control and disease modification or biomarkers of treatment-resistant epilepsy.

## Supplementary Material

fcad355_Supplementary_Data

## Data Availability

RNA-sequencing data from studies using iPSCs were deposited into Gene Expression Omnibus and are available under the accession number GSE211696. The iCLIP data are deposited under accession GSE214317. The human brain small RNA-sequencing data are available at the European Genome-phenome Archive (EGA) under the accession number EGAS00001003922. Scripts for differential miRNA editing used in this analysis were deposited to GitHub (https://github.com/NgocTNguyen/mirna-differential-editing-analysis). All other data are available in the main paper or through cited references.
