# Supplementary material for: Differential microRNA editing may drive target pathway switching in human temporal lobe epilepsy
Source: Brain Commun. 2024 Jan 3;6(1):fcad355. doi: 10.1093/braincomms/fcad355 (PMC10781512; doi:10.1093/braincomms/fcad355)
Supplement: fcad355_Supplementary_Data [file fcad355_supplementary_data.zip › Supplementary figures.pdf]

Supplementary Figure 1

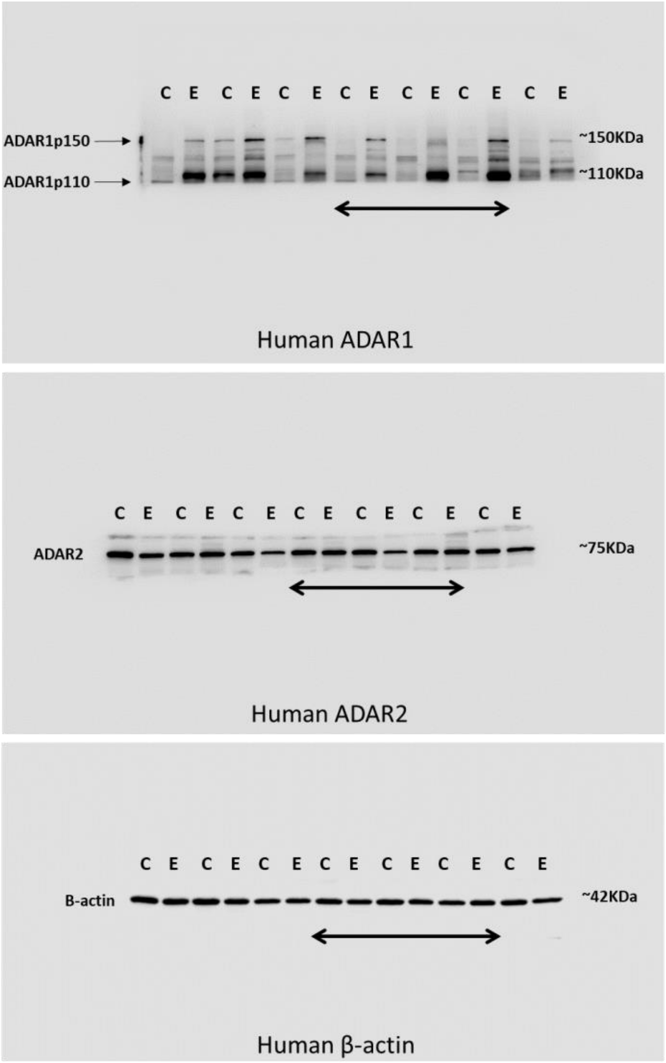

Supplementary Fig. 1. Uncropped immunoblots for Adenosine deaminase acting on RNAs (ADARs) in the hippocampus from temporal lobe epilepsy patients

Uncropped western blots for Figure 1 showing ADAR1 (top), ADAR2 (middle), and β-actin (bottom) using hippocampal samples from autopsy controls (C) and TLE patients (E). β-actin is shown as a guide to loading. Arrows at the bottom of the immunoblots indicate the region presented in the main figure (membrane were cut to enable parallel incubation of different antibodies).

Supplementary Figure 2

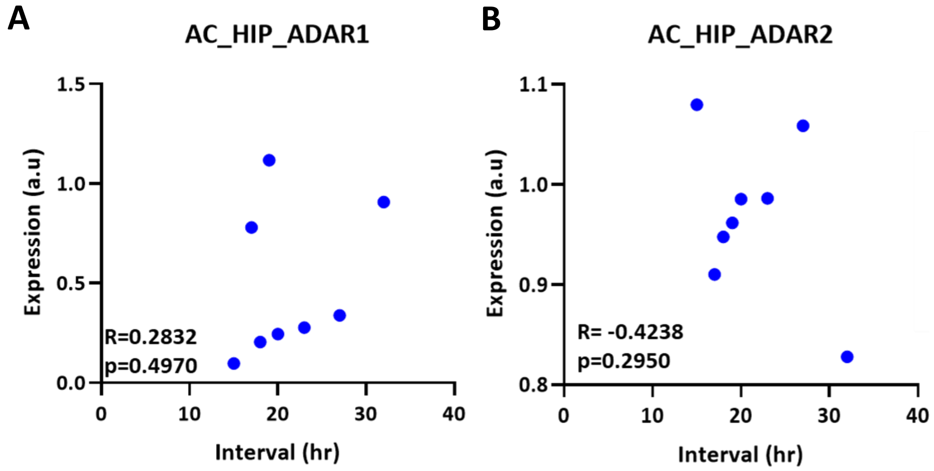

Supplementary Fig. 2. Effects of post-mortem interval on protein levels of Adenosine deaminase acting on RNAs (ADARs)

Pearson correlation coefficient was used to plot the correlation of **(A)**, ADAR1 and **(B)**, ADAR2, protein levels against post-mortem interval for the hippocampus (HIP) from the autopsy controls (AC). Analysis indicates that there is no significant correlation between the duration of post-mortem interval and protein levels for both ADAR proteins (n = 8).

Supplementary Figure 3.

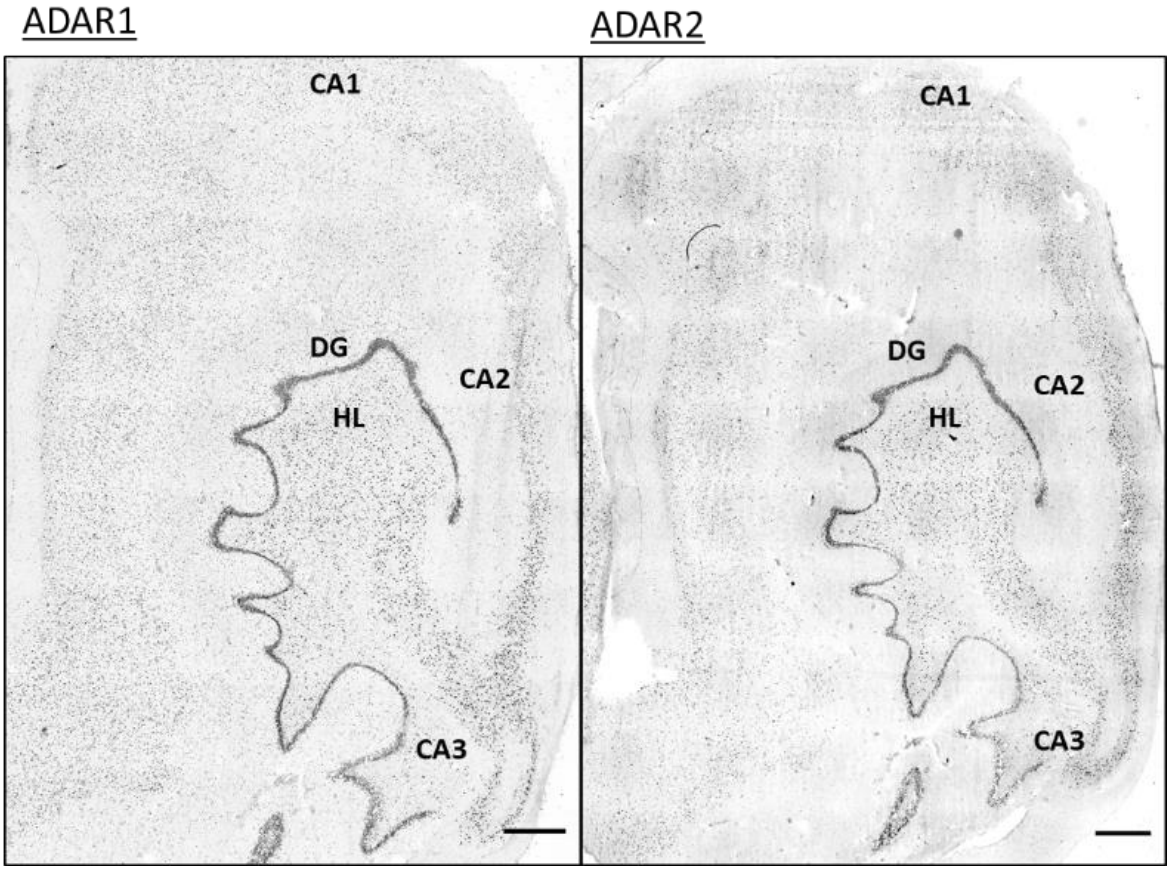

Supplementary Fig. 3. Hippocampal Adenosine deaminase acting on RNAs (ADAR1 and ADAR2) immunostaining in human autopsy control

Representative staining of (*left*) ADAR1 and (*right*) ADAR2 in different sub-fields (CA1, CA2, CA3, DG and HL) of the hippocampus from an autopsy control (scale bar 1000 µm, total magnification of 40x. CA: Cornu Ammonis, DG: Dentate gyrus, HL: Hilus). Both ADARs can be observed in all sub-fields of the hippocampus and are most notable in neuron-rich layers.

Supplementary Figure 4

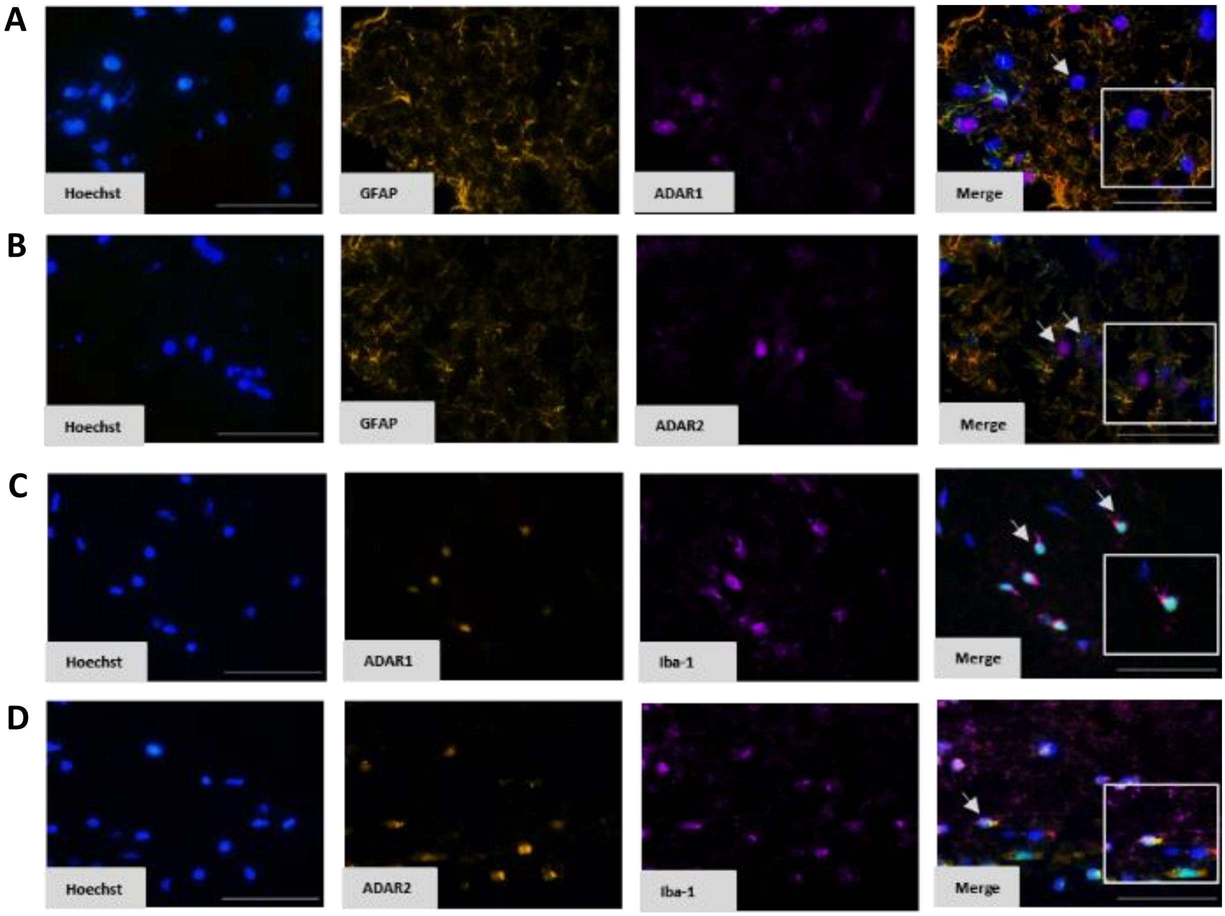

Supplementary Fig. 4. Non-neuronal adenosine deaminase acting on RNA (ADAR1 and ADAR2) immunostaining in human temporal lobe epilepsy

Representative immunostaining showing limited **(A)**, ADAR1 (magenta) or **(B)**, ADAR2 (magenta) signals overlap with glial fibrillary acidic protein (GFAP, yellow). Similar limited overlapping signals can be seen between **(C)**, ADAR1 (yellow) or **(D),** ADAR2 (yellow) with ionized calcium-binding adapter molecule 1 (Iba-1, magenta). Hoechst dye (blue) was used as nuclear stain for DNA (scale bar 50 µm, total magnification of 1000x with oil immersion). White arrowheads indicate the presence of overlapping signals, and the magnified version is shown in the white box of each panel.

Supplementary Figure 5

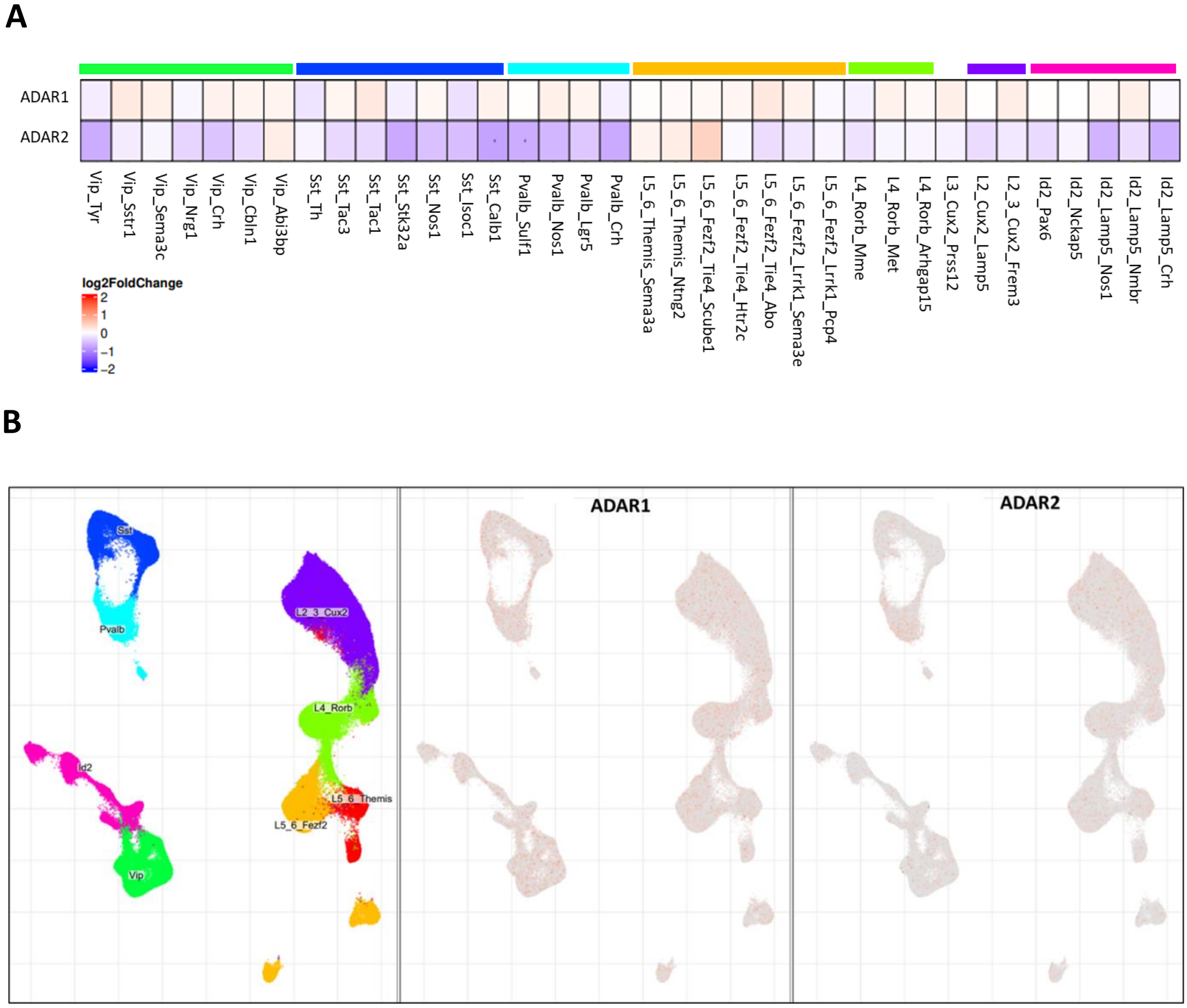

Supplementary Fig. 5. Single-nucleus RNA sequencing analysis of human Adenosine deaminase acting on RNA (ADAR) family expression across multiple neuronal subtypes

(A) Differential expression (log2Foldchange) of ADAR family members (*ADAR1/ADAR*; *ADAR2/ADARB1*) between human epilepsy and control samples across 36 neuronal subtypes.[1] Statistical significance with FDR < 0.05 is marked by *. Colour scheme on x-axis indicate the panels representing different neuronal subtypes. **(B)** UMAP representation of nuclei from human epilepsy and control temporal cortex. The colours in the first panel represent different neuronal subtypes as identified in a previous study conducted by Pfisterer and colleagues. Red and/or grey colour scale is proportional to log-normalized expression values across single cells.

Supplementary Figure 6

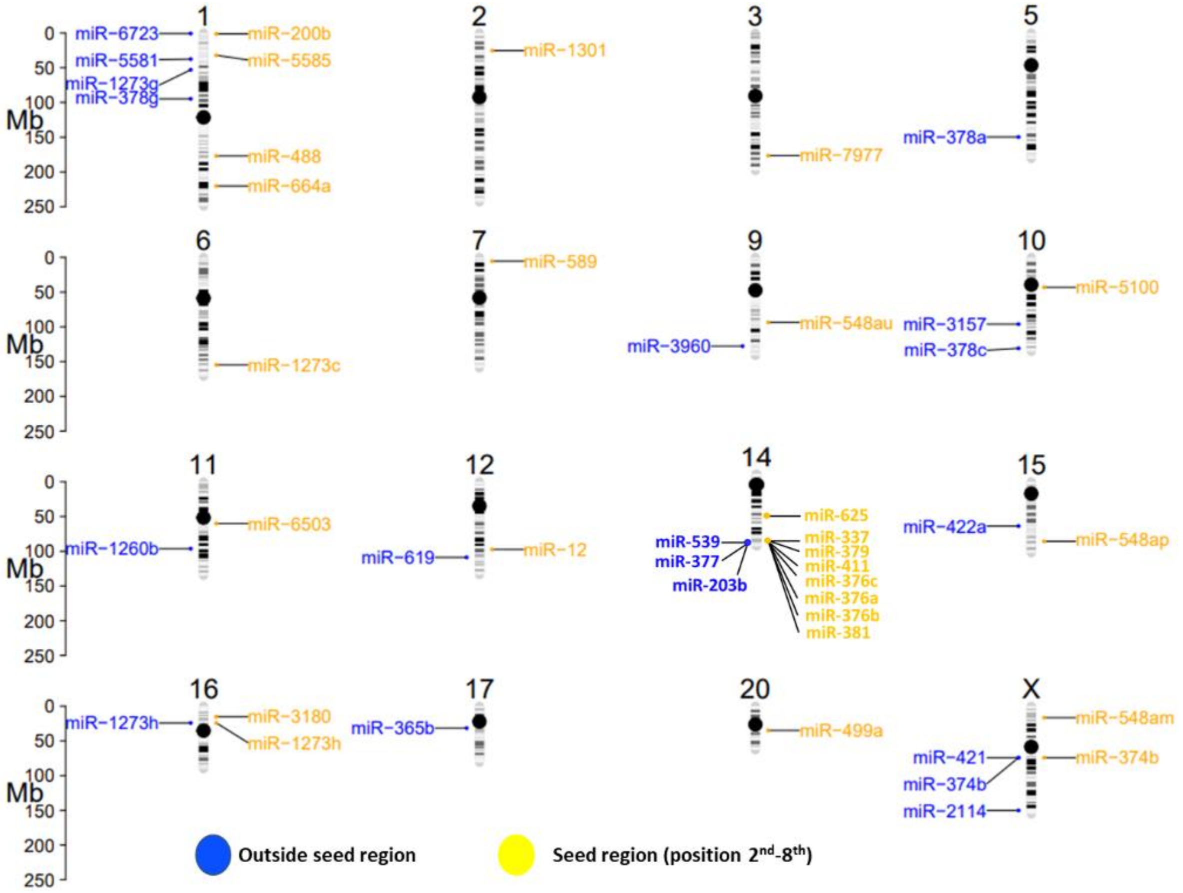

Supplementary Fig. 6. MicroRNA (miRNA) editing footprint across chromosomes

Graphic displays locations of miRNA genes in which editing was observed in the hippocampus of human autopsy controls and temporal lobe epilepsy patients. Chromosome 1 and 14 are enriched with miRNAs displaying likely ADAR editing. Layers above or below the centromere representing the p and q arm of a chromosome, respectively. Chromosomes without ADAR-mediated editing in miRNAs are not shown. MiRNAs are colour-coded with blue to indicate editing outside of the seed region, while yellow indicates editing occurs within the seed region (position 2nd to 8th of the mature sequence). (n = 8 controls, 16 epilepsy).

Supplementary Figure 7

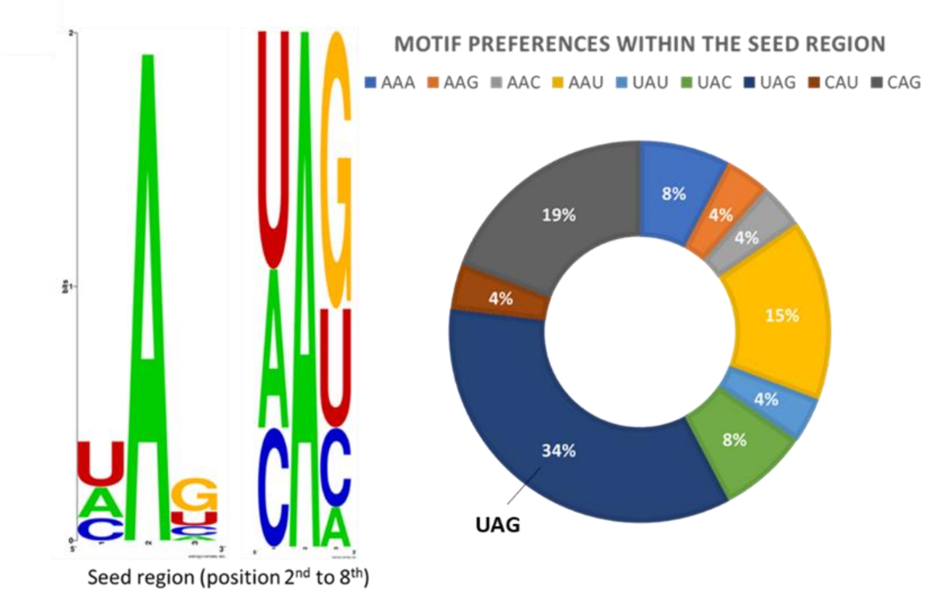

Supplementary Fig. 7. Motif analysis of microRNA (miRNA) editing sites within the seed region of miRNAs in the hippocampus of autopsy controls and temporal lobe epilepsy patients

Graphic illustrates the sequence motifs within the seed region up- and down-stream of the miRNA editing sites targeted by adenosine deaminase acting on RNA (ADARs). The frequency of adenosine from the triplet of 5’ -UAG- 3’ motif targeted by ADARs is the highest within the seed region. Sequence motifs were generated from WebLogo v. 2.8.2.

Supplementary Figure 8

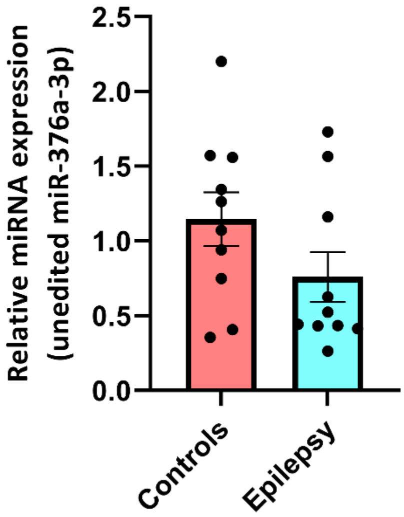

Supplementary Fig. 8. Levels of non-edited form of miR-376a-3p in the hippocampus of control and temporal lobe epilepsy (TLE) patients

Graph showing Taqman miRNA assay analysis of the relative expression levels of the non-edited miR-376a-3p in the hippocampus of a validation cohort of controls and patients with TLE (Mean ± SEM, n = 10/group, Ordinary two-tailed T test; non-significant).

Supplementary Figure 9

A

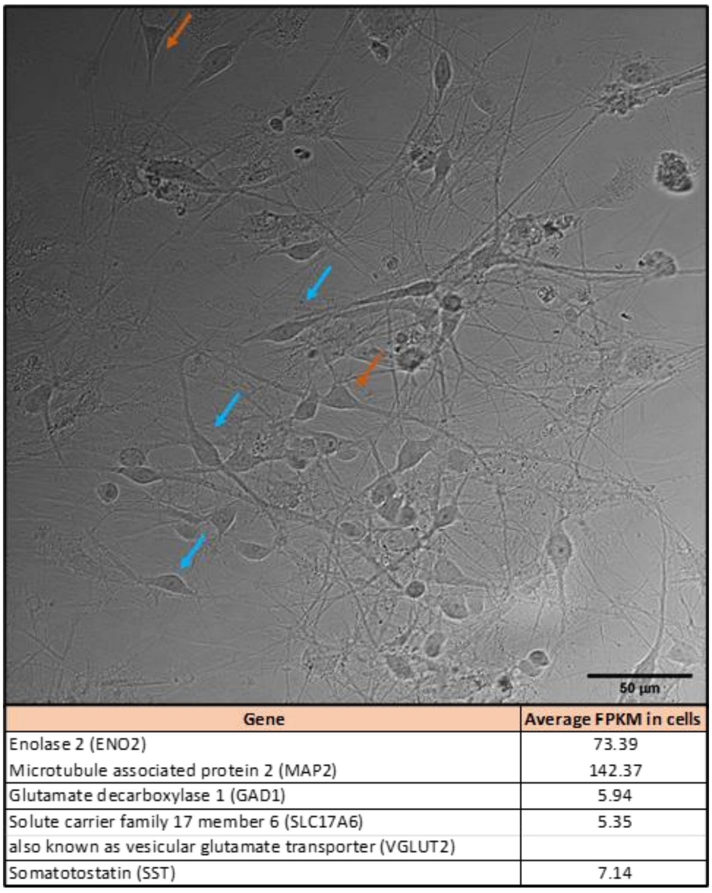

Gene	Average FPKM in cells
Enolase 2 (ENO2)	73.39
Microtubule associated protein 2 (MAP2)	142.37
Glutamate decarboxylase 1 (GAD1)	5.94
Solute carrier family 17 member 6 (SLC17A6)	5.35
also known as vesicular glutamate transporter (VGLUT2)	
Somatotostatin (SST)	7.14

B C

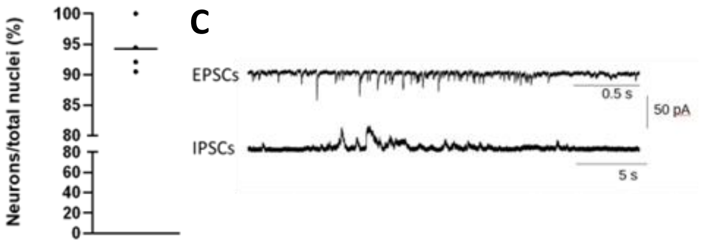

Supplementary Fig. 9. Characterisation of human induced pluripotent stem cell (iPSC)-derived neurons 30-day post-differentiation

(A) Representative field view of human iPSC-derived neurons in which different subtypes can be observed, including bipolar (blue arrowhead) and pyramidal (orange arrowhead) neurons. (Scale bar 50 µm, total magnification 100x). The table below show the average Fragments Per Kilobase of transcript per Million mapped reads (FPKM) for mature neuron markers, *ENO2* and *MAP2*, inhibitory neuron markers, *GAD1* and *SST*, and excitatory neuron marker, *VGLUT2* sequenced from the neurons. **(B)** Percentage of cells double-positive for neuronal marker TUJ1 and nuclei stain Hoechst. **(C)** Representative voltage clamp recording showing excitatory postsynaptic currents (EPSCs) at a holding potential of −70 mV (top) or inhibitory postsynaptic currents (IPSCs) at 0 mV (bottom) confirming mixed excitatory and inhibitory neurons.

Supplementary Figure 10

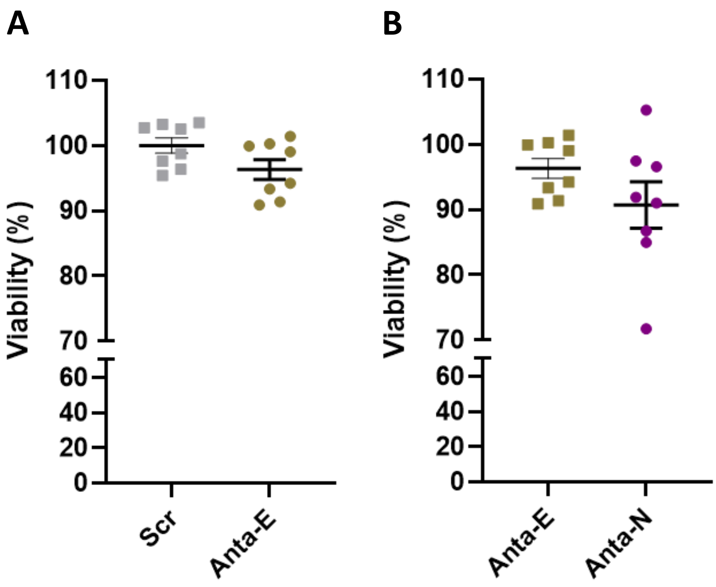

Supplementary Fig. 10. Viability of human neurons treated with antimirs targeting miR-376a-3p

(A) Viability was measured using media tested with WST-1 solution. Graph shows comparison of the viability between neurons treated with scramble (Scr) and antimirs targeting the edited miR-376a-3p (Anta-E). Both treatment groups showed comparable viability percentage in cells. **(B)** Graph showing viability of neurons treated with Anta-E and antimirs targeting the non-edited-miR-376a-3p (Anta-N). Values from scramble-treated neurons were used as normalisation (Mean ± SEM, n = 8 per treatment group, Ordinary two-tailed T test; non-significant).

Supplementary Figure 11

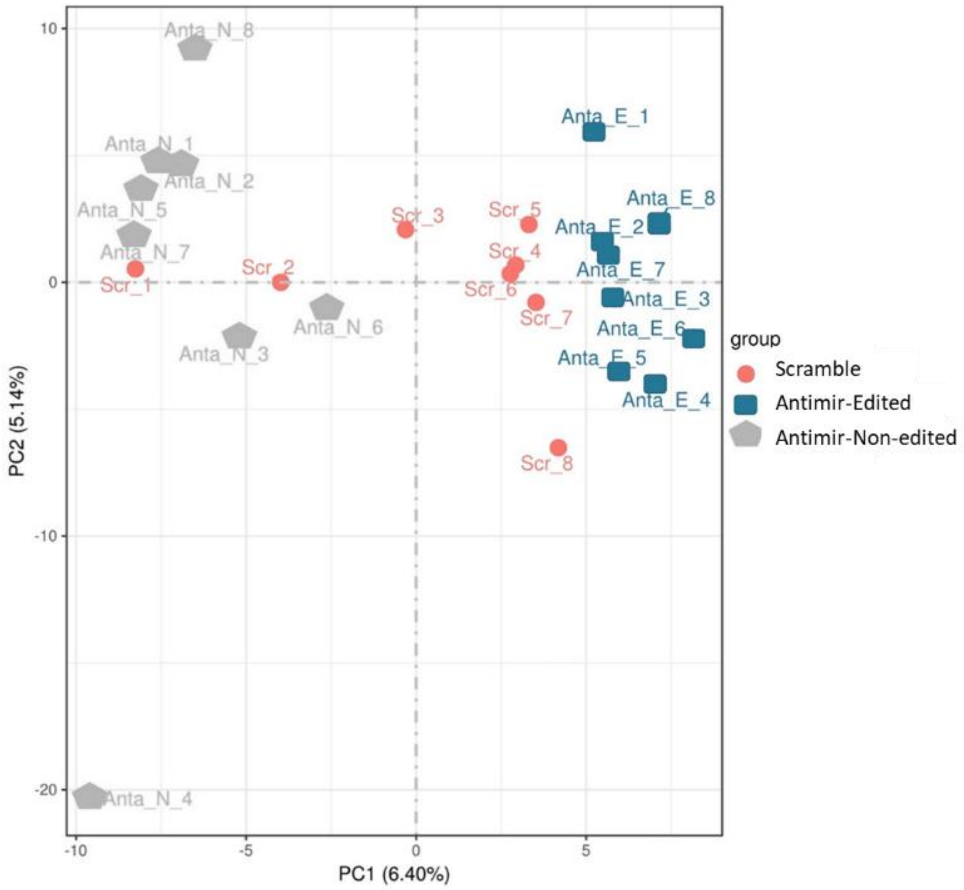

Supplementary Fig. 11. Effects of antimir targeting of miR-376a-3p on gene expression

Principle component analysis (PCA) of the gene expression profiles from different treatment groups. Anta-N, antimir-Non-edited; Anta-E, antimir-Edited; Scrm, scramble. (n = 8 per group). Note, targeting the edited and unedited forms produces largely distinct clustering of samples.

Supplementary Figure 12

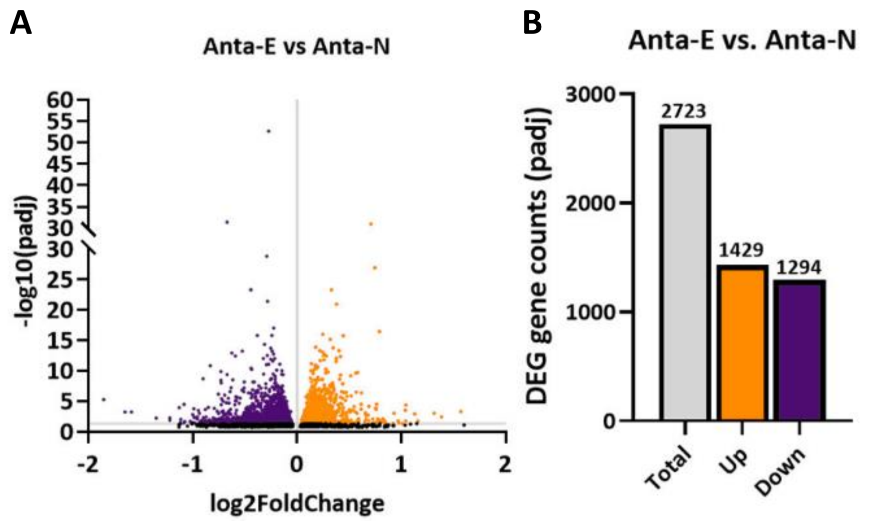

Supplementary Fig. 12. Differentially expressed genes (DEGs) between neurons treated with antimir-Edited (Anta-E) vs. antimir-Non-edited (Anta-N)

(A) Volcano plot and **(B)** bar graph showing the values of DEGs between Anta-E vs. Anta-N. (n=8 per treatment group).

Supplementary Figure 13

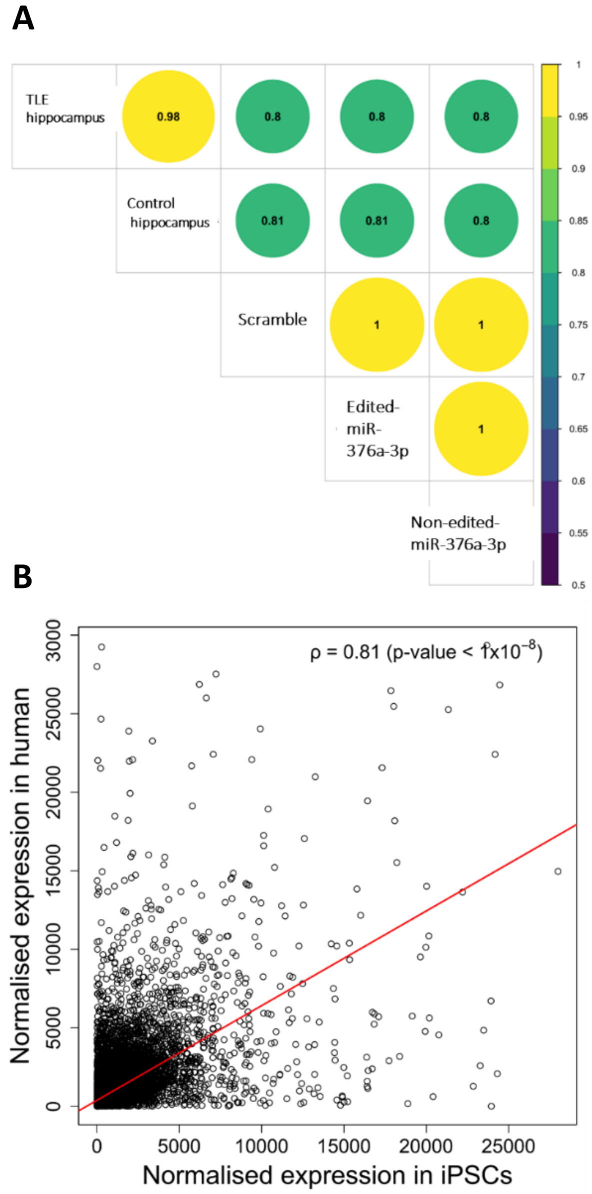

Supplementary Fig. 13. Correlation between RNA-sequencing data from induced pluripotent stem cell (iPSC) and human brain

(A) Spearman's rank correlation coefficient was calculated between the normalised expression of the expressed genes from RNA-sequencing data from the hippocampus of control and individuals with temporal lobe epilepsy and iPSC-derived neurons treated with a scrambled antimir, antimir targeting the edited form of miR-376a-3p and antimir targeting the non-edited form of miR-376a-3p. All correlations were significant p < 1x10^-8. **(B)** Spearman's rank correlation coefficient between the normalised expression of the expressed genes in human hippocampal and iPSC RNA-sequencing data, regardless of condition. (n=8 per treatment group).

References for Supplementary Figures

1. Pfisterer U, Petukhov V, Demharter S*, et al*. Identification of epilepsy-associated neuronal subtypes and gene expression underlying epileptogenesis. *Nat Commun*. 2020;11(1):5038. doi:10.1038/s41467-020-18752-7
